# Baseline neutrophil-to-eosinophil-ratio and outcome in metastatic clear-cell renal cell carcinoma treated with nivolumab or ipilimumab/nivolumab

**DOI:** 10.2340/1651-226X.2024.40390

**Published:** 2024-08-11

**Authors:** Yana Beulque, Lisa Kinget, Eduard Roussel, Sajedeh Mobaraki, Annouschka Laenen, Philip R Debruyne, Yannick Van Herck, Marcella Baldewijns, Agnieszka Wozniak, Abhishek D. Garg, Jessica Zucman-Rossi, Gabrielle Couchy, Maarten Albersen, Liesbeth De Wever, Lorenz Haaker, Benoit Beuselinck

**Affiliations:** a Department of General Medical Oncology, University Hospitals Leuven, Leuven, Belgium; b Department of Urology, University Hospitals Leuven, Leuven, Belgium; c Biostatistics and Statistical Bioinformatics Center, Leuven, Belgium; d Department of General Medical Oncology, AZ Groeninge, Kortrijk, Belgium; e Medical Technology Research Centre (MTRC), School of Life Sciences, Anglia Ruskin University, Cambridge, UK; f School of Nursing and Midwifery, University of Plymouth, Plymouth, UK; g Department of Pathology, University Hospitals Leuven, Leuven, Belgium; h Laboratory of Experimental Oncology, KU Leuven, Leuven, Belgium; i Laboratory of Cell Stress & Immunity (CSI), Department of Cellular & Molecular Medicine, KU Leuven, Leuven, Belgium; j Inserm, UMRS-1138, Génomique Fonctionnelle Des Tumeurs Solides, Centre de Recherche des Cordeliers, Paris, France; k Department of Radiology, University Hospitals Leuven, Leuven, Belgium; l Department of Medical Oncology, AZ Sint Lucas, Brugge, Belgium

**Keywords:** Renal cell carcinoma, neutrophil-to-eosinophil ratio, checkpoint inhibitor, immunotherapy, biomarker

## Abstract

**Background and purpose:**

This study aims to evaluate neutrophil-to-eosinophil ratio (NER) as a prognostic and/or predictive biomarker in metastatic clear cell renal cell carcinoma (m-ccRCC) treated with nivolumab or ipilimumab/nivolumab.

**Patients/materials and methods:**

We performed a retrospective study on m-ccRCC patients treated with nivolumab or ipilimumab/nivolumab (2012–2022). Baseline NER was calculated and correlated with clinical outcomes: response rate (RR), progression free survival (PFS) and overall survival (OS). Corresponding transcriptomic data were analysed.

**Results:**

We included 201 m-ccRCC patients, 76 treated with ipilimumab/nivolumab and 125 with nivolumab. Baseline NER was statistically significantly associated with International Metastatic RCC Database Consortium (IMDC) risk groups. Increased NER was associated with shorter PFS and OS in the total patient series and nivolumab-treated patients. In patients treated with ipilimumab/nivolumab, increased NER was only statistically significantly associated with shorter OS. The impact of baseline NER on PFS and OS was independent of IMDC risk stratification. No clear correlation was found between baseline NER and RECIST response or maximal tumour shrinkage. In two additional databases, NER was also associated with PFS and OS in first-line vascular-endothelial-growth-factor-receptor tyrosine-kinase-inhibitors (VEGFR-TKIs), but not to disease-free survival in the post-nephrectomy setting. Lower NER was associated with intratumoural molecular features possibly associated with better outcome on immune checkpoint inhibitors.

**Interpretation:**

Lower baseline NER is associated with better PFS and OS, independent of IMDC risk score, in m-ccRCC patients treated with ipilimumab/nivolumab or nivolumab. It correlates with intratumoural molecular features possibly associated with better outcome on immune checkpoint inhibitors. The predictive power of this biomarker is probably limited and insufficient for patient selection.

## Introduction

In recent years, therapeutic options for metastatic RCC (mRCC) have expanded with vascular-endothelial-growth-factor-receptor tyrosine-kinase-inhibitors (VEGFR-TKIs) and immune checkpoint inhibitors (ICIs) [1]. Currently used ICIs include programmed death 1 (PD-1) inhibitors nivolumab and pembrolizumab, the programmed death ligand 1 (PD-L1) inhibitor avelumab, and the cytotoxic T-lymphocyte-associated antigen 4 (CTLA-4) inhibitor ipilimumab. First-line strategies involve the double ICI-combination ipilimumab/nivolumab or combinations with VEGFR-TKIs (cabozantinib/nivolumab, axitinib/pembrolizumab, lenvatinib/pembrolizumab) [2]. Nivolumab monotherapy is used in later lines after VEGFR-TKIs.

The International Metastatic RCC Database Consortium (IMDC) prognostic score, developed in the era of VEGFR-TKIs, remains pivotal in patient risk stratification [3]. The score correlates with outcomes under ICIs, guiding the selection of first-line therapies [4]. Ipilimumab/nivolumab is approved only for IMDC intermediate/poor risk, while VEGFR-TKIs/ICI-combinations are also approved for IMDC good risk patients [5–7]. The IMDC score incorporates performance status, time to therapy initiation, hypercalcemia, anaemia, thrombocytosis, and neutrophilia [3], reflecting disease aggressiveness and tumour-induced inflammation. Other inflammatory markers like elevated neutrophil-to-lymphocyte ratio (NLR) and CRP, have been associated with worse outcomes in mRCC patients treated with ICIs [8, 9]. Neutrophil-to-eosinophil ratio (NER) shows promise as a prognostic marker across several cancers treated with ICIs: in melanoma, non-small-cell lung cancer and head-and-neck cancer higher baseline eosinophils and/or lower NER correlate with better outcomes on ICIs [10–12]. In mRCC, three retrospective studies indicate that lower baseline NER may predict improved OS on ICIs [13–15]. Two of them also described an impact on PFS and overall RR.

This study investigates the impact of baseline NER on PFS/OS in m-ccRCC patients treated with nivolumab or ipilimumab/nivolumab. Additionally, we assess NER in first-line VEGFR-TKI and post-nephrectomy, and analyse transcriptomic data.

## Patients and methods

M-ccRCC patients who started nivolumab or ipilimumab/nivolumab between 2012 and 2022 at the University Hospitals in Leuven and the General Hospital Groeninge (Kortrijk) were enrolled. Ethical approval was obtained from the hospital ethics committees.

We collected data about demographics, IMDC risk score, clinical outcomes and laboratory values (neutrophils, lymphocytes, eosinophils). NER and NLR were calculated by dividing absolute neutrophil count by absolute eosinophil or lymphocyte count.

The primary endpoint, OS, was measured from ICI initiation to death. PFS served as a secondary endpoint, due to complexities in its evaluation considering atypical responses (mixed response, pseudoprogression) [16]. PFS was calculated from ICI-initiation to radiographic progression based on iRECIST (immune Response Evaluation Criteria in Solid Tumours) [17] or death, whichever occurred first. We evaluated maximal tumour shrinkage and best iRECIST response: partial response (PR), stable disease (SD), or progressive disease (PD).

Post-nephrectomy, we correlated NER with disease-free-survival (DFS; time between nephrectomy with curative intent and the development of metastases or local recurrence) in a random sample series of patients who underwent nephrectomy with curative intent. In patients treated with first-line VEGFR-TKIs, we studied the impact on PFS/OS. Only patients treated before the ICI era were included, to avoid confounding effects of nivolumab on OS in later lines.

Univariate and multivariate Cox proportional hazards models were used for the analyses of PFS/OS/DFS. Results are reported as hazard ratios (HR) with 95% confidence intervals. The proportional hazards assumption was verified using graphic plots of Schoenfeld residuals over time. Log-transformations were applied to deal with skewness. Continuous variables were employed for Cox proportional hazards models, while dichotomized values were utilized for graphical representation via Kaplan–Meier estimates. For selecting the optimal cut-off value, all possible dichotomizations (≤cut-off/>cut-off) for NER were considered. Cut-off values were ordered in terms of model fit and the value that leads to the highest likelihood was selected. Harrel C-index was used to quantify and compare the discriminative value between NER and IMDC versus IMDC alone for OS. Analyses were conducted using SAS software (version 9.4).

RNA extraction, sequencing and subsequent analysis of transcriptomic data followed established methodologies as prescribed previously [18]. For immune cell population estimates, the online CIBERSORTx platform was used (https://cibersortx.stanford.edu) with LM22 as signature matrix, using absolute mode. IMmotion150 [19], Javelin101 [20], and tumour low HLA promiscuity (tLHP) [21] signatures were calculated as previously reported. For Gene Set Enrichment Analysis (GSEA), the GSEA JAVA software was used (v4.1.0; Hallmark, Reactome, ImmuneSigDB, and Gene Ontology gene sets). Non-parametric tests (i.e. Spearman correlation and Mann–Whitney U test) were performed given the non-normal distribution of (log-)NER and (log-)NLR (as evaluated by Shapiro–Wilk test). Correction for multiple testing was performed using the Benjamini–Hochberg method (False discovery rate [FDR]) as indicated. Statistical analysis and visualization were performed in GraphPad and R (v. 4.2.1).

## Results

### Included patients

A total of 201 m-ccRCC patients were included, 76 treated with ipilimumab/nivolumab and 125 with nivolumab (Patient demographics: Table 1). Most patients treated with nivolumab monotherapy received it in second or later line, although five patients (4%) received it in first-line. Baseline demographics were similar across therapeutic subgroups.

**Table 1:** Included patients.

Patient characteristics	Overall			Nivolumab	Ipilimumab/ Nivolumab
	ALL	NER ≤ median	NER > median		
Number of patients	201	101	100	125	76
Age at start of ICI, years, median (range)	67 (31–90)	66 (31–90)	68 (44–88)	67 (31–88)	66.5 (44–90)
Gender, *n* (%)					
Male	149 (74)	71 (71)	77 (77)	92 (74)	57 (75)
Female	52 (26)	29 (29)	23 (23)	33 (26)	19 (25)
ECOG performance status^a^, *n* (%)					
0	121 (60)	76 (75)*	45 (45)*	66 (53)	55 (73)
1	64 (32)	21 (21)*	43 (43)*	44 (35)	20 (26)
≥ 2	16 (8)	4 (4)*	12 (12)*	15 (12)	1 (1)
Previous VEGFR, *n* (%)	120 (60)	59 (58)	61 (61)	120 (96)	0 (0)
ICI as first-line therapy, *n* (%)	81 (40)	42	39	5 (4)	76 (100)
ICI as second-line therapy, *n* (%)	87 (43)	44	43	87 (70)	-
ICI as third-line therapy, *n* (%)	26 (13)	14	12	26 (21)	-
ICI as fourth-line therapy and beyond, *n* (%)	7 (3)	1	6	7 (6)	-
Fuhrman grade, *n* (%)					
Grade 1	2 (1)	0 (0)	2 (2)	2 (2)	0 (0)
Grade 2	29 (14)	16 (16)	13 (13)	18 (14)	11 (14)
Grade 3	75 (37)	40 (40)	35 (35)	48 (38)	27 (36)
Grade 4	80 (40)	42 (41)	38 (38)	52 (42)	28 (37)
Unknown	15 (8)	3 (3)	12 (12)	5 (4)	10 (13)
Sarcomatoid dedifferentiation, *n* (%)					
0%	124 (79)	68 (80)	56 (79)	84 (82)	40 (75)
1–24%	28 (18)	14 (16)	14 (20)	19 (18)	9 (17)
25% or more	4 (3)	3 (4)	1 (1)	0	4 (8)
IMDC risk group^b^, *n* (%)					
Good	27 (13)	19 (19)**	8 (8)**	15 (12)	12 (16)
Intermediate	125 (62)	69 (68)**	56 (56)**	76 (61)	49 (64)
Poor	49 (25)	13 (13)**	36 (36)**	35 (27)	15 (20)
*Metastatic sites, *n* (%)					
Lymph nodes	102 (51)	46 (46)	56 (56)	63 (50)	39 (51)
Lung	124 (62)	64 (63)	60 (60)	76 (61)	48 (63)
Liver	44 (22)	21 (21)	23 (23)	31 (25)	13 (17)
Brain	14 (7)	2 (2)	12 (12)	13 (10)	1 (1)
Bone	83 (41)	39 (39)	44 (44)	57 (46)	26 (34)
Pancreas	28 (14)	14 (14)	14 (14)	23 (18)	5 (7)
Other	120 (60)	58 (57)	62 (62)	77 (62)	43 (57)
Number of metastatic sites, median (range)	3 (1–7)	2 (1–8)	3 (1–7)	3 (1–8)	2 (1–6)
Baseline lab values					
Neutrophil/mm³, median (range)	4249 (442–15832)	3651 (442–8182)	4874 (1788–15832)	4003 (442–13707)	4645.5 (2413–15832)
Eosinophils/mm³, median (range)	122 (7–1974)	182 (29–1974)	68.5 (7–167)	108 (9–1974)	133.5 (7–607)
Lymphocytes/mm³, median (range)	1288 (192–3772)	1318 (270–3772)	1277 (192–3242)	1238 (192–3772)	1361 (348–3671)
Baseline NER^c^, median (range)	33.8 (1.6–1583.2)	–	–	35.9 (1.6–1370.7)	32.7 (5.5–1583.2)
Baseline NLR^c^, median (range)	3.3 (0.5–30.4)	3.0 (0.5–17)	3.8 (1.6–30.4)	3.4 (0.5–30.4)	3.3 (1.0–18.7)

ICI: immune checkpoint inhibition; VEGFR: vascular endothelial growth factor receptor; ECOG: Eastern Cooperative Oncology Group; IMDC: International Metastatic RCC Database Consortium; NER: neutrophil-to-eosinophil ratio; NLR: neutrophil-to-lymphocyte ratio.

a Eastern Cooperative Oncology Group classification ranging from 1 to 5, with lower scores indicating better functionality.

b International Metastatic RCC Database Consortium used to risk-stratify disease within good, intermediate, and poor risk prognostic groups.

* Chi Square *p* < 0.0001.

** Chi Square *p* = 0.0002.

Median baseline NER was 33.8 (range 1.6–1583.2) and median baseline was NLR 3.3 (range 0.5–30.4), which similar across both treatment groups. Patients with NER >median, had worse ECOG performance status (*p* = 0.0002) and were more often IMDC poor risk (*p* < 0.0001). Median baseline NER differed across IMDC risk groups (*p* < 0.001) (Supplementary Figure 1). There was no correlation between baseline NER and sarcomatoid dedifferentiation, a well-known unfavourable prognostic factor [22, 23]. Baseline neutrophils and eosinophils or lymphocytes showed no correlation using linear regression, but eosinophils were positively correlated to lymphocytes (*p* = 0.001) and NER to NLR (*p* < 0.0001) (Supplementary Figure 2).

### NER

#### Univariate analysis

In a continuous analysis, increased NER was associated with shorter PFS/OS in the entire cohort (*p* = 0.002 for PFS; *p* < 0.0001 for OS) and in the nivolumab subgroup (*p* = 0.004 for PFS; *p* < 0.0001 for OS). In the ipilimumab/nivolumab subgroup, increased NER was associated with shorter OS (*p* = 0.002), but the association with PFS was not statistically significant (*p* = 0.23) (Table 2).

**Table 2:** Results of univariate analysis in patients treated with nivolumab and ipilimumab/nivolumab.

Test		HR (95% CI) for PFS	*P*	HR (95% CI) for OS	*P*
**All patients (*n* = 201)**					
Neutrophils	×2 units	1.29 (1.03; 1.62)	0.03	1.54 (1.18; 2.00)	0.002
Eosinophils	×2 units	0.88 (0.78; 0.99)	0.03	0.75 (0.66; 0.85)	<0.0001
Lymphocytes	×2 units	0.98 (0.79; 1.22)	0.86	0.75 (0.60; 0.95)	0.02
NER	×2 units	1.17 (1.06; 1.29)	0.002	1.33 (1.20; 1.47)	<0.0001
NLR	×2 units	1.20 (1.01; 1.44)	0.04	1.56 (1.28; 1.89)	<0.0001
IMDC	Global test		0.001		<0.0001
	Good vs. Poor	0.54 (0.32; 0.92)	0.02	0.25 (0.13; 0.50)	<0.0001
	Intermediate vs. Poor	0.52 (0.36; 0.74)	0.0004	0.37 (0.25; 0.56)	<0.0001
**NIVOLUMAB (*n* = 125)**					
Neutrophils	×2 units	1.48 (1.13; 1.93)	0.004	1.69 (1.21; 2.37)	0.002
Eosinophils	×2 units	0.90 (0.78; 1.04)	0.14	0.79 (0.67; 0.92)	0.003
Lymphocytes	×2 units	1.01 (0.80; 1.29)	0.91	0.81 (0.62; 1.07)	0.14
NER	×2 units	1.18 (1.06; 1.33)	0.004	1.32 (1.16; 1.51)	<0.0001
NLR	×2 units	1.27 (1.05; 1.53)	0.01	1.52 (1.22; 1.90)	0.0002
IMDC	Global test		0.05		0.0003
	Good vs. Poor	0.58 (0.30; 1.14)	0.11	0.30 (0.13; 0.70)	0.005
	Intermediate vs. Poor	0.59 (0.38; 0.92)	0.02	0.40 (0.26; 0.65)	0.0002
**IPILIMUMAB/NIVOLUMAB (*n* = 76)**					
Neutrophils	×2 units	1.27 (0.76; 2.12)	0.37	1.49 (0.90; 2.46)	0.12
Eosinophils	×2 units	0.89 (0.70; 1.13)	0.33	0.69 (0.55; 0.87)	0.002
Lymphocytes	×2 units	1.18 (0.73; 1.89)	0.50	0.72 (0.55; 1.16)	0.18
NER	×2 units	1.13 (0.93; 1.37)	0.23	1.32 (1.11; 1.56)	0.002
NLR	×2 units	1.02 (0.69; 1.51)	0.94	1.60 (1.07; 2.40)	0.02
IMDC	Global test		0.02		0.005
	Good vs. Poor	0.52 (0.22; 1.24)	0.14	0.20 (0.06; 0.73)	0.02
	Intermediate vs. Poor	0.38 (0.20; 0.75)	0.005	0.33 (0.16; 0.69)	0.003

HR: hazard ratio; CI: confidence interval; PFS: progression free survival; OS: overall survival; IMDC: International Metastatic RCC Database Consortium; NER: neutrophil-to-eosinophil ratio; NLR: neutrophil-to-lymphocyte ratio.

HR reflects the relative change in risk of progression or death for a doubling (×2 units) of the predictor value.

**Figure 1:**
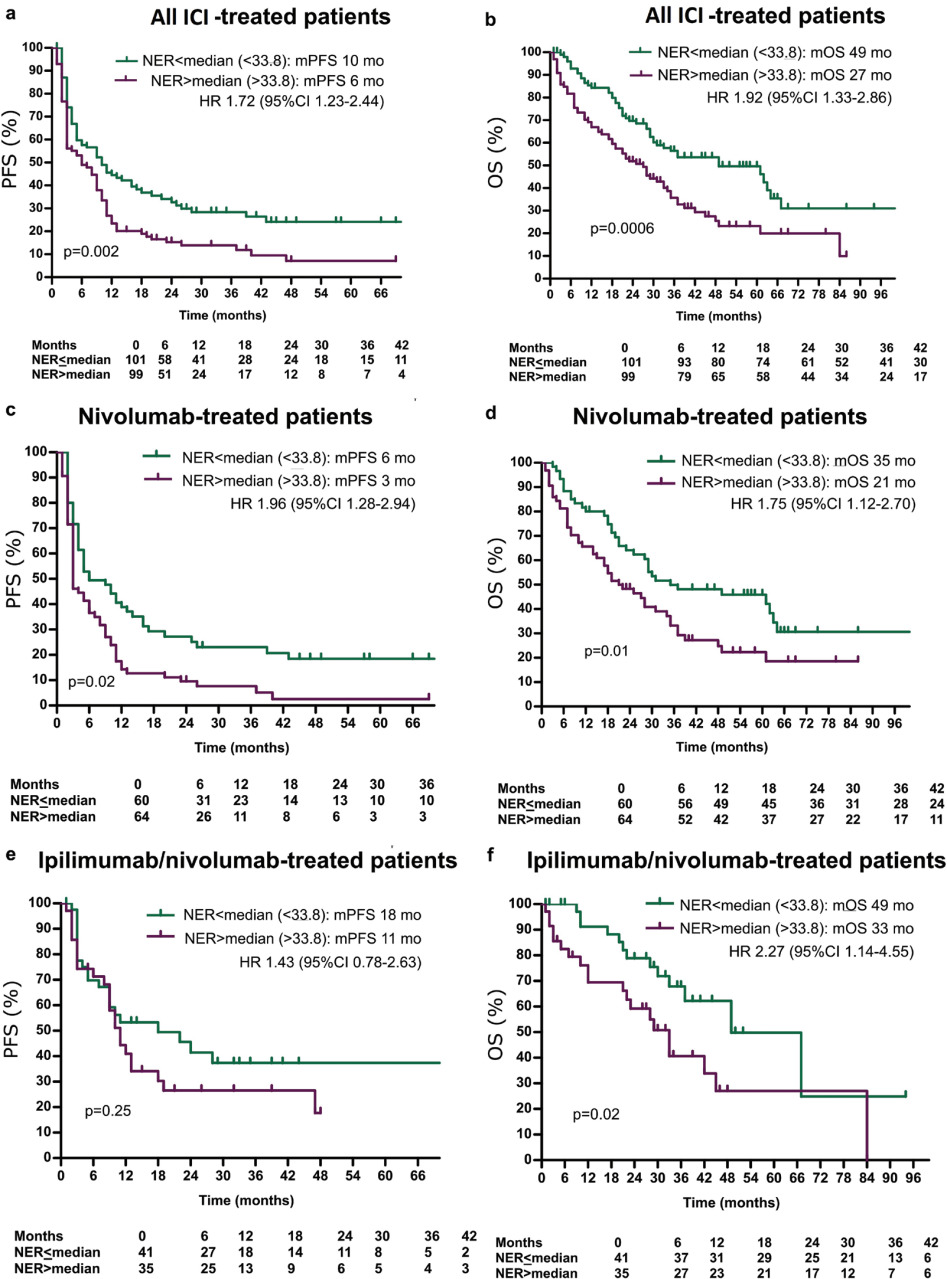
(a–f) Kaplan–Meier estimates of the impact of NER on progression-free survival and overall survival in all patients treated with immunotherapy.

The optimal NER cut-off for PFS/OS was close to the median NER value. Hence we used the median for dichotomized analysis. Patients with NER ≤ 33.8 had a longer median PFS (mPFS) (10 months vs. 6 months; *p* = 0.002; Figure 1a) and median OS (mOS) (49 months vs. 27 months; *p* = 0.0006; Figure 1b). Similar trends were observed in both subgroups (Figure 1c–f).

**Figure 2:**
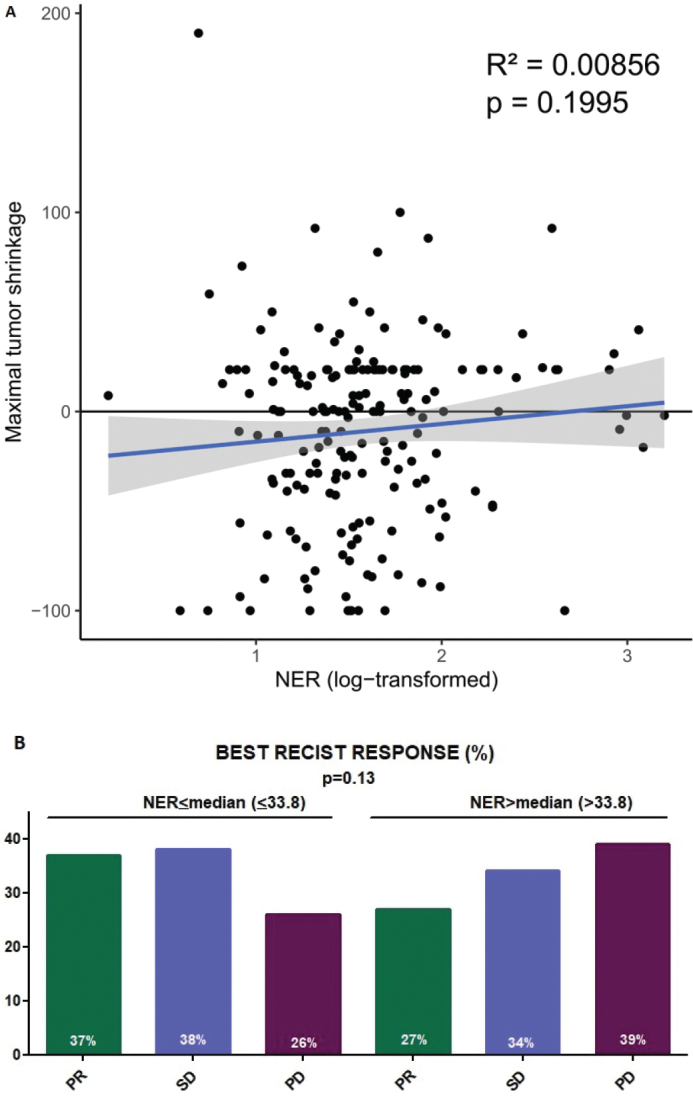
(a) Correlation between best tumour shrinkage and NER. (b) Best PECIST response in different NER subgroups. PR: partial response; SD: stable disease; PD: progressive disease.

Tumour response, as measured by maximal tumour shrinkage and RECIST best response, did not correlate with baseline NER levels (*p* = 0.20 and *p* = 0.13, respectively) (Figure 2a, b).

#### Multivariable analysis

After adjusting for NLR, ICI type and IMDC risk groups, increased NER remained associated with shorter PFS (*p* = 0.04) and OS (*p* = 0.003). IMDC risk groups were also independently associated with PFS (*p* = 0.01) and OS (*p* = 0.0002), while NLR was not. In both the treatment subgroups, NER and IMDC risk groups maintained their independent correlation with OS (Table 3). Supplemental Figure 3 shows NER’s distinctive potential across all IMDC risk groups.

**Table 3:** Results of multivariable analysis in patients treated with nivolumab and ipilimumab/nivolumab.

Test		HR (95% CI) for PFS	*P*	HR (95% CI) for OS	*P*
**All patients for baseline NER (*n* = 201)**					
First line TT	**Nivolumab**-**Ipilimumab** vs. **Nivolumab**	0.55 (0.39; 0.78)	0.0007	0.71 (0.47; 1.07)	0.10
NER	×2 units	1.14 (1.01; 1.28)	0.04	1.21 (1.07; 1.38)	0.003
NLR	×2 units	1.01 (0.82; 1.26)	0.90	1.18 (0.93; 1.51)	0.18
IMDC	Global test		0.01		0.0002
	Good vs. Poor	0.63 (0.36; 1.11)	0.11	0.37 (0.18; 0.77)	0.008
	Intermediate vs. Poor	0.56 (0.39; 0.82)	0.003	0.44 (0.29; 0.66)	<0.0001
**All patients for eosinophils (*n* = 201)**					
Eosinophils	×2 units	0.92 (0.81; 1.04)	0.18	0.83 (0.72; 0.95)	0.007
NLR	×2 units	1.08 (0.90; 130)	0.42	1.30 (1.06; 1.60)	0.01
IMDC	Global test		0.01		0.0002
	Good vs. Poor	0.61 (0.35; 1.06)	0.08	0.38 (0.18; 0.77)	0.008
	Intermediate vs. Poor	0.56 (0.38; 0.82)	0.003	0.45 (0.30; 0.67)	<0.0001
**Nivolumab for baseline NER (*n* = 125)**					
NER	×2 units	1.13 (0.98; 1.30)	0.10	1.18 (1.01; 1.37)	0.04
NLR	×2 units	1.09 (0.85; 1.38)	0.51	1.24 (0.95; 1.63)	0.11
IMDC	Global test		0.16		0.007
	Good vs. Poor	0.68 (0.34; 1.37)	0.28	0.45 (0.19; 1.07)	0.07
	Intermediate vs. Poor	0.65 (0.41; 1.01)	0.06	0.47 (0.29; 0.75)	0.002
**Ipilimumab/Nivolumab for baseline NER (*n* = 76)**					
NER	×2 units	1.18 (0.90; 1.55)	0.22	1.33 (1.02; 1.75)	0.04
NLR	×2 units	0.66 (0.39; 1.10)	0.11	0.88 (0.48; 1.59)	0.67
IMDC	Global test		0.01		0.03
	Good vs. Poor	0.39 (0.14; 1.07)	0.07	0.24 (0.06; 0.95)	0.04
	Intermediate vs. Poor	0.32 (0.15; 0.69)	0.003	0.36 (0.16; 0.81)	0.01

HR: hazard ratio; CI: confidence interval; PFS: progression free survival; OS: overall survival; IMDC: International Metastatic RCC Database Consortium; NER: neutrophil-to-eosinophil ratio; NLR: neutrophil-to-lymphocyte ratio; TT: Treatment type.

HR reflects the relative change in risk of progression or death for a doubling (×2 units) of the predictor value.

Combined analysis of NER and NLR, showed highest mOS in NER and NLR low patients (63 months), while OS was similar in the three other subgroups (Supplementary Figure 4). Adding NER or NLR to IMDC risk improved the accuracy to predict OS (Harrel C-index increased from 0.64 to 0.68 and from 0.64 to 0.67, respectively; Supplementary Table 1).

### Eosinophils

Higher baseline eosinophil counts were associated with improved PFS (*p* = 0.03) and OS (*p* < 0.0001) (Table 2). Dichotomized analysis showed a longer mPFS (10 months vs. 6 months; *p* = 0.04) and mOS (49 months vs. 28 months; *p* = 0.007) (Supplementary Figure 5A, B) for patients with baseline eosinophils above median (>122). Similar impact on PFS/OS was observed in the subgroup of patients treated with ipilimumab/nivolumab and nivolumab as monotherapy. After adjusting for NLR and IMDC risk, baseline eosinophil count remained associated with OS (*p* = 0.007), but not PFS (Table 3). Adding it to IMDC risk improved the accuracy to predict OS (Harrel-C index increased from 0.64 to 0.68), similar to the impact of NER (Supplementary Table 1).

### Neutrophils and NLR

Elevated baseline neutrophil counts predicted shorter PFS (*p* = 0.03) and OS (*p* = 0.002), while baseline lymphocyte counts correlated positively with OS (*p* = 0.02), but not PFS. IMDC risk groups were associated with both PFS (*p* = 0.001) and OS (*p* < 0.0001). Increased NLR was associated with shorter PFS (*p* = 0.04) and OS (*p* < 0.0001) (Table 2). Dichotomized results are shown in Supplemental Figure 6.

### Post-nephrectomy setting

Out of 315 patients, 96 eventually relapsed, while 219 had not at the time of data cut-off. Continuous analysis (Supplementary Table 2) and Kaplan–Meier estimates showed no impact of NER on DFS post-nephrectomy. NLR was statistically significantly associated with DFS post-nephrectomy. Baseline eosinophil counts were not associated with DFS post-nephrectomy (Supplementary Figure 7A–C).

### First-line VEGFR-TKIs

**Figure 3:**
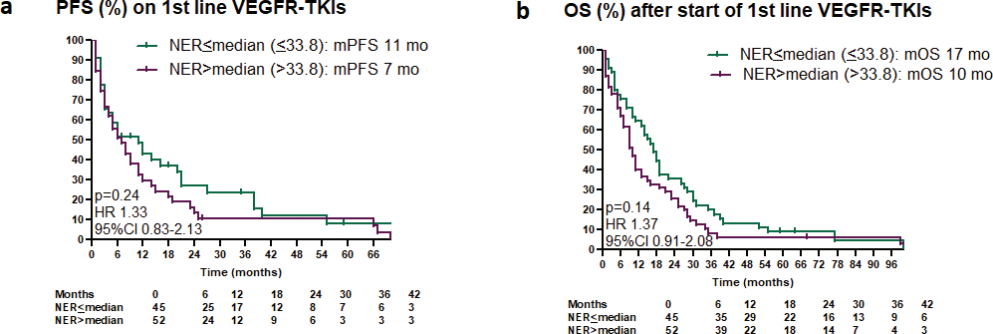
(a, b) Kaplan–Meier estimates of the impact of NER on progression-free survival and overall survival after start of VEGFR-tyrosine kinase inhibitors.

In a cohort of 100 patients, 71 received sunitinib and 29 pazopanib. Increased baseline NER was associated with shorter PFS (*p* = 0.007) and OS (*p* = 0.005). Bivariate analysis including IMDC risk groups found no independent correlation between NER and PFS (*p* = 0.11) or OS (*p* = 0.08). Kaplan–Meier estimates for PFS/OS based on median NER levels are illustrated in Figure 3a, b. ORR and maximal tumour shrinkage were not significantly associated with NER (Supplementary Figure 8). Baseline eosinophil counts were not associated with PFS/OS on first-line VEGFR-TKIs (Supplementary Figure 9 A, B) (Supplementary Table 2).

### NER and eosinophil evolution between baseline and week 6

For this analysis data of 169 patients were available. Overall, median NER declined from 33.4 (baseline) to 26.7 (week 6) (*p* = 0.09): it decreased from 31.9 to 21.5 in patients with PR (*p* = 0.053), from 33.2 to 26.5 in those with SD (*p* = 0.36) and remained stable (40.0 to 40.7; *p* = 0.61) in those with early PD as best response. The change of NER between baseline and week 6 did not correlate with PFS/OS (Supplementary Table 3), but NER levels at week 6 did correlate with both PFS (*p* = 0.0006) and OS (*p* < 0.0001) (Supplementary Figure 10A, B).

Median eosinophil count increased from 128 to 153 (*p* = 0.01). The increase was statistically significant in the PR subgroup (129 to 194; *p* = 0.03), but not in patients with SD (130 to 158; *p* = 0.16) and early PD (102 to 121; *p* = 0.16). The change of eosinophils between baseline and week 6 did not correlate with PFS/OS (Supplementary Table 3), but absolute eosinophil count at week 6 did correlate with PFS (*p* = 0.0003) and OS (*p* < 0.0001) (Supplementary Figure 10C, D).

### Differences in tumour microenvironment

**Figure 4:**
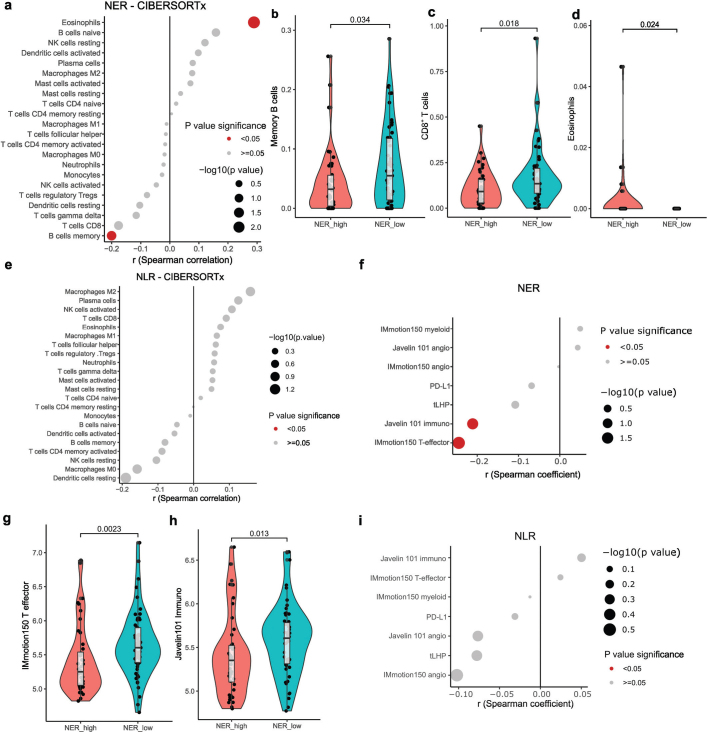
Transcriptomic correlates of neutrophil-eosinophil ratio (NER) and neutrophil-lymphocyte ratio (NLR) at the level of the primary tumour. (A) Dotplot showing correlation coefficient and p values of Spearman correlation between NER and tumour immune cell estimates (CIBERSORTx). (B–D) Violin- and boxplots showing memory B cells (b), CD8^+^ T cells (c) and eosinophils (d) as deconvoluted by CIBERSORTx, between NERLOW and NERHIGH groups. (E) Dotplot showing correlation coefficient and p values of Spearman correlation between NLR and tumour immune cell estimates (CIBERSORTx). (F) Dotplot showing correlation coefficient and p values of Spearman correlation between NER and PD-L1 (by CD274 expression), tLHP and Javelin101 and IMmotion150 gene signatures. (G–H) Violin- and boxplots showing IMmotion150 T effector signature (g) and Javelin101 Immuno signature (h), between NERLOW and NERHIGH groups. (I) Dotplot showing correlation coefficient and p values of Spearman correlation between NER and PD-L1 (by CD274 expression), Javelin101 and IMmotion150 gene signatures.

Differences in NER, baseline eosinophil counts and NLR at the start of ICIs were correlated with transcriptomic features of the tumour microenvironment in 99 primary tumours. We deconvoluted tumour-infiltrating immune cell populations (CIBERSORTx). NER was correlated inversely with memory B-cells and positively with increased eosinophils (Figure 4a). Dichotomized NER showed higher CD8^+^ T-cell infiltration in NER-low tumours, alongside consistent results for memory B-cells and eosinophils (Figure 4b–d). With FDR-correction, these results did not remain statistically significant. Baseline eosinophil counts had comparable results: higher peripheral counts correlated with gamma/delta T-cells and memory B-cells, albeit non-significantly after FDR-correction (Supplementary Figure 11A). NLR analysis yielded no statistically significant results (Figure 4e).

We checked gene expression signatures from biomarker analyses of the Javelin101 and IMmotion150 trials and the tLHP signature which was recently developed as an ICI biomarker for ccRCC, and PD-L1 (by *CD274* expression) [19–21]. NER correlated inversely with Javelin101 Immuno and IMmotion150 T-effector signatures, albeit not statistically significant after FDR-correction (Figure 4f). When dichotomized, both IMmotion150 T-effector and Javelin101 Immuno signatures were higher in NER-low tumours (Figure 4g, h; statistically significant after FDR-correction). These results were consistent with higher CD8^+^ T-cell infiltration. There were was a statistically non-significant correlation with tLHP, and PD-L1 expression. IMmotion150 myeloid signature, linked to ICI resistance, had statistically non-significant enrichment in tumours with higher NER. Baseline eosinophil counts again showed comparable patterns, where a correlation with the IMmotion150 T-effector and Javelin101 Immuno signatures was observed (Supplementary Figure 11B; statistically significant after FDR-correction). For NLR, a statistically non-significant enrichment of angiogenesis-related signatures was seen in NLR-low tumours (Figure 4i).

Finally, GSEA compared NER-high versus NER-low samples, exploring Hallmark, Reactome, Immunesigdb, and Gene Ontology genesets. While statistical significance was not reached (FDR *q* < 0.25), NER-low samples showed enrichment in interferon pathways, PD1-signalling, MHC-class-II presentation, and co-stimulation, often linked to favourable ICI outcomes. Comparatively, NER-high tumours had enrichment of TGFb-pathways, MYC-targets, and other implied in ICI resistance/tumour aggressiveness (Supplementary Figure 12). Similar results were observed for samples with low versus high baseline eosinophil counts (Supplementary Figure 11C–F). NLR-low samples showed distinct patterns, notably enrichment of different angiogenesis pathways, implying a more indolent tumour phenotype (Supplementary Figure 13).

## Discussion

This study aimed to investigate the prognostic and/or predictive impact of baseline NER on PFS and OS in m-ccRCC patients treated with nivolumab or ipilimumab/nivolumab. Additionally, we assess NER in first-line VEGFR-TKI and post-nephrectomy, and analyse transcriptomic data.

In 201 m-ccRCC patients treated with ICI, increased NER was associated with shorter PFS/OS, independent of IMDC risk stratification. NER improved IMDC prognostication accuracy (increased Harrel C-index). No correlation was found between NER and RECIST response or maximal tumour shrinkage. NER did not impact DFS post-nephrectomy, but did correlate with PFS/OS on first-line VEGFR-TKIs. Baseline eosinophil count was also associated with PFS/OS in ICI treated patients, with a similar performance as NER, but not with PFS/OS on VEGFR-TKIs or with DFS post-nephrectomy. NER-low tumours showed CD8^+^ T-cell and immune-related pathway enrichment, indicating favourable immune activation of the tumour micro-environment. In contrast, NLR-low tumours had a trend towards higher angiogenesis, implying a more indolent tumour phenotype.

### Parallel findings in literature

Three other studies have explored the impact of NER on outcome in mRCC patients treated with ICIs (Supplementary Table 4). On 110 m-ccRCC patients treated with ipilimumab/nivolumab, avelumab or pembrolizumab, patients with baseline NER < median (<26.4) had a higher ORR (40% vs. 21.8%; *p* = 0.04), longer mPFS (8.6 months vs. 3.2 months; *p* < 0.01) and mOS (not reached vs. 27.3 months; *p* < 0.01) [13]. For 184 mRCC patients treated with ipilimumab/nivolumab, using a higher NER cut-off (49.2), with 25% of patients in the NER-high group, elevated NER was associated with lower OS (*p* = 0.048), but not with PFS nor ORR [14]. On 49 m-RCC patients treated with nivolumab (cut-off 48), median PFS and OS were shorter in NER-high patients (3 months vs. 30 months; *p* < 0.001 and 6 months vs. 24 months; *p* = 0.002, respectively). NER correlated with ORR: 87.5% in NER-low compared to 12.5% in NER-high patients (*p* = 0.003) [15].

Recently, NER value was noted in 442 patients treated with the combination of the ICI avelumab and the VEGFR-TKI axitinib in the Javelin Renal 101 phase III trial [24]. Lower NER (as continuous variable) was associated with improved PFS and OS. RR was 63.9% in patients with NER < median and 55.2% in patients with NER > median. The impact of NER was also studied in 444 sunitinib treated patients: similar to our results, PFS and RR were not impacted by NER. However, OS was longer in patients with lower NER, but at least a third of patients were treated with ICIs in second or later line [25], whereas those patients were excluded in our analysis.

### Predictive and/or prognostic impact

Three previous studies and our study link lower NER-levels with improved OS in mRCC patients receiving ICI therapy. The correlation with PFS/ORR appears to be less robust. Two studies described an increased RR in NER-low patients [13, 15], implying a predictive value. In our study, NER did not statistically significantly affect RR or tumour shrinkage. However, response evaluation in patients treated by ICIs is difficult, due to atypical responses such as pseudoprogression [16]. Moreover, response evaluation may be underestimated. In a neoadjuvant trial in melanoma, anatomopathological examination on resected metastases showed a pathological complete response in many cases, while on imaging best response was a limited PR or SD or even an early PD [26].

In our study, NER showed no association with DFS post-nephrectomy, indicating no prognostic impact, whereas NLR did demonstrate a clear association with DFS post-nephrectomy. We did however observe an association of NER with PFS and OS in first-line VEGFR-TKIs treatment, but not with ORR, indicating a prognostic impact.

Taken together, baseline NER is clearly associated with PFS/OS on ICIs, indicating a prognostic value. With conflicting data on association with RR, the predictive power – if present – is probably limited and insufficient for patient selection.

### NLR

Our research aligns with recent studies on baseline NLR levels in various metastatic cancers treated with ICIs, including mRCC. Elevated NLR is consistently associated with worse survival [8, 9]. Our study is not powered to compare the prognostic impact of NER and NLR.

In VEGFR-TKI treated mRCC, two studies investigated the prognostic significance of NLR. On multivariable analysis in 45 patients, low NLR (≤ 2) was significantly associated with longer mPFS (23.9 months vs. 8.6 months; *p* = 0.04) [27]. On 439 patients treated with sunitinib, patients with NLR < median had a longer mPFS and mOS, and a better RR. Within the IMDC poor-risk group, there was a high frequency of patients with NLR at or above the median [28].

In 786 patients with localized RCC, pre-operative NLR > median (>2.7) was associated with higher recurrence rates at 3 (24% vs. 6%) and 5 years (37% vs. 12%) [29].

### Evolution of NER during ICI therapy

Recently, in 150 mRCC patients, a correlation between a decrease in NER at week 6 after ICI initiation and improved PFS/OS was found [30]. Two studies investigated the evolution of eosinophils during ICI therapy. In 65 mRCC patients, an increase in eosinophils at week six of nivolumab was associated with a better outcome, independently of IMDC risk factors [31]. In 264 mRCC patients treated with nivolumab, on-treatment increase in eosinophils by week 8 predicted improved PFS/OS [32]. We could not validate these findings in our patient series.

### Transcriptomic data

Limited data exists on intratumoural molecular correlates of NLR. Higher NLR was associated with the expression of Hallmark pathway signatures of MYC-target, co-expression of cell-cycle, epithelial-to-mesenchymal transition – markers of more aggressive RCC. Lower NLR was associated with less chromosomal instability and a higher prevalence of the polybromo-1 mutation, both markers of indolent disease. An association between median NLR and expression of cell type–specific signatures for M0- and M2-macrophages and resting CD4 memory T-cells was found on deconvolution. The 26-gene Javelin101 immune signature was associated with below-median NLR. No relationship between NLR and the Javelin101 and IMmotion150 angiogenesis signature was observed. Neither PD-L1–positive expression (1% threshold), nor the presence of CD8^+^ T-cells, was associated with differences in NLR [20, 28]. In a second study, NLR-low patients had more CD8^+^ and CD4^+^ T-cell infiltration in their tumours [33], a surprising finding, as CD8^+^ T-cell infiltration is a marker of poor outcome in mRCC [34].

Data on intratumoural correlates of NER are scarce. Our data should be validated in independent and larger patient series. Despite inconclusive findings, distinct microenvironmental features were observed in NER-low versus NLR-low tumours, suggesting potential influences on ICI response. NER-low tumours showed CD8^+^ T-cell enrichment and Javelin101 Immuno and IMmotion150 T-effector signatures, as well as pro-inflammatory pathways. Comparatively, enrichment of gamma-delta T-cells was observed in both NER-low and eosinophil-high tumours, possibly reflecting an underlying chronic, unsupervised inflammation immune phenotype. In contrast, NLR-low tumours had a trend towards higher angiogenesis (both Javelin101 and IMmotion150 angiogenesis signatures, as well as GSEA pathways), implying a more indolent tumour phenotype. Very recently, in a dichotomized analysis (<or>median NER value) on data of the phase III trial comparing avelumab/axitinib with sunitinib, NER could not be associated with Javelin101 and Immotion150 signatures [24].

## Conclusion

Lower baseline NER is associated with better PFS/OS, independent of IMDC risk score in m-ccRCC patients treated with ipilimumab/nivolumab or nivolumab. It correlates with intratumoural molecular features possibly associated with better outcome on ICIs. The predictive power of this biomarker is probably limited and insufficient for patient selection. Prospective validation is needed.

## Disclosures

PRD reports leader- and ownership of Dr. Philip Debruyne (BV); received grants (to institution) from Pfizer; received consulting fees for participation on advisory boards from Astellas Pharma, BMS, Ipsen, Merck, and Pfizer; received honoraria for lectures from Bayer; received travel support from Janssen; serves as a (substitute) board member for the Clinical Trials College, Federal Public Service, Kingdom of Belgium; and holds stock in Alkermes, Mural Oncology PLC and Biocartis Group NV. B.B. reports speaker’s fee from BMS, Pfizer, MSD and Ipsen, consulting fees for participation on advisory boards from BMS, Ipsen and MSD, and an unrestricted research grant from BMS.

## Data availability statement

Data are available upon request at the corresponding author.

## Ethics declaration and trial registry information

Ethical approval was obtained from the hospital ethics committees (S53479 and S63833). Data were treated according to GDPR legislation. Patients signed an informed consent allowing the use of the anonymized data.

## Supplementary Material




