# Supplementary material for: Baseline neutrophil-to-eosinophil-ratio and outcome in metastatic clear-cell renal cell carcinoma treated with nivolumab or ipilimumab/nivolumab
Source: Acta Oncol. 2024 Aug 11;63:40390. doi: 10.2340/1651-226X.2024.40390 (PMC11332468; doi:10.2340/1651-226X.2024.40390)

Supplementary material has been published as submitted. It has not been copyedited, or typeset by Acta Oncologica

SUPPLEMENTAL FIGURE 1: ASSOCIATION OF NER AND IMDC RISK GROUPS. COMPARISON AND P-VALUE AS CALCULATED BY ANOVA.

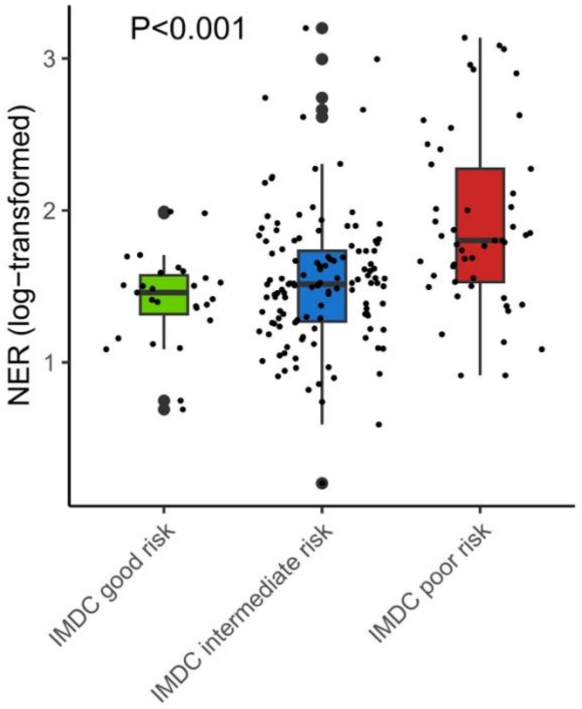

SUPPLEMENTAL FIGURE 2: ASSOCIATION OF LYMPHOCYTES, EOSINOPHILS, NEUTROPHILS, NER AND NLR

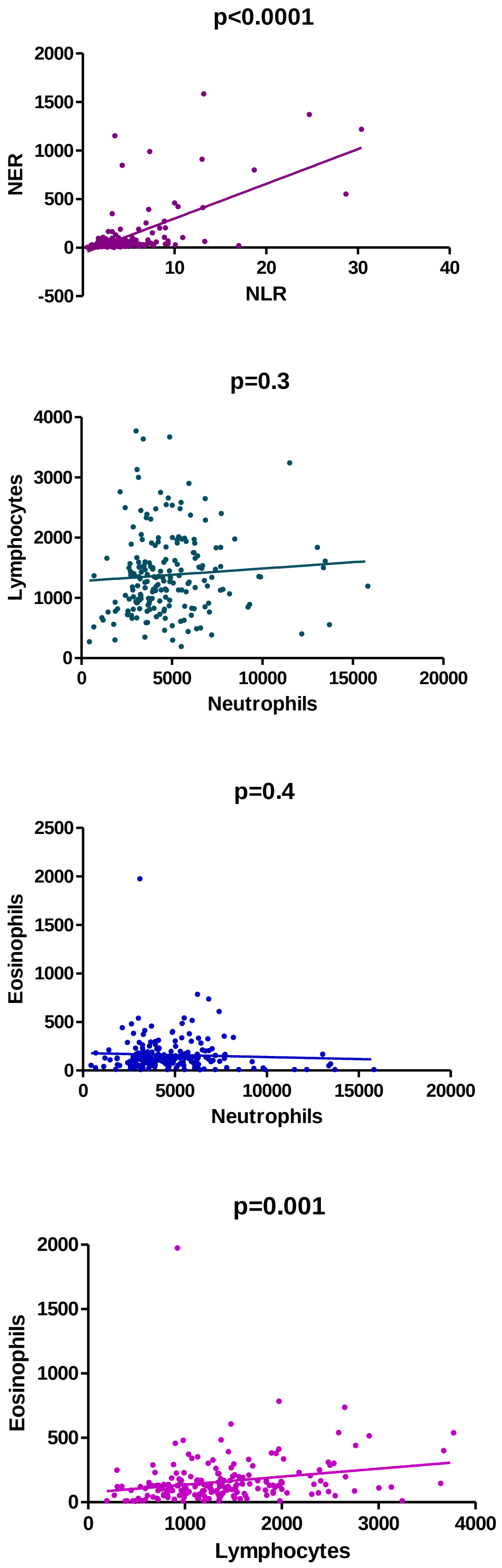

SUPPLEMENTAL FIGURE 3: KAPLAN-MEIER ESTIMATES OF THE IMPACT OF NER AND IMDC RISK GROUPS ON OVERALL SURVIVAL

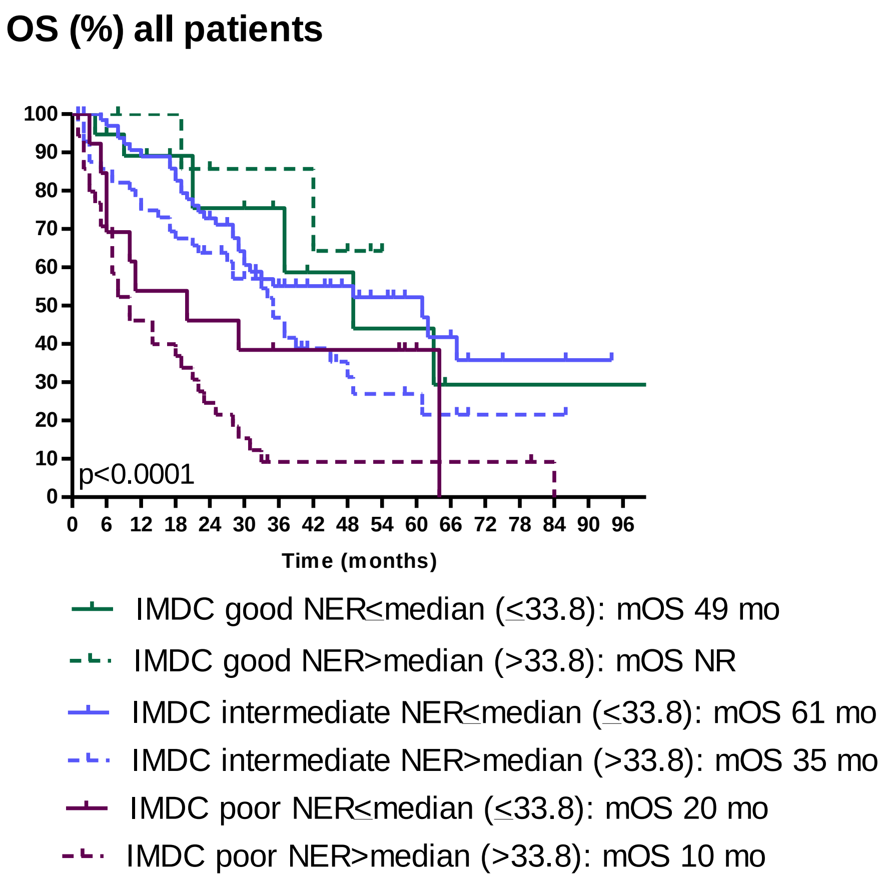

SUPPLEMENTAL FIGURE 4: KAPLAN-MEIER ESTIMATES OF THE IMPACT OF NER AND NLR ON OVERALL SURVIVAL

OS (%) all patients

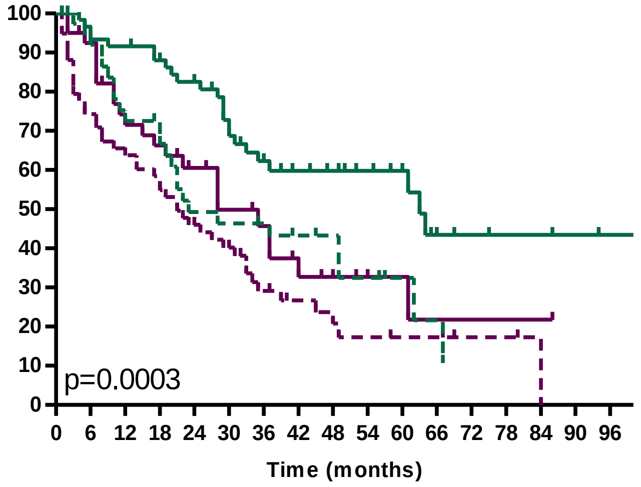

NLR≤median (≤3.3) NER>median (>33.8): mOS 28 mo

NLR>median (>3.3) NER>median (>33.8): mOS 21 mo

NLR≤median (≤3.3) NER≤median (≤33.8): mOS 63 mo

NLR>median (>3.3) NER≤median (≤33.8): mOS 23 mo

SUPPLEMENTAL FIGURE 5: PANEL A-B: KAPLAN-MEIER ESTIMATES OF THE IMPACT OF EOSINOPHILS AND NEUTROPHILS ON PROGRESSION-FREE SURVIVAL AND OVERALL SURVIVAL IN ALL PATIENTS TREATED WITH IMMUNOTHERAPY

PFS (%) all patients

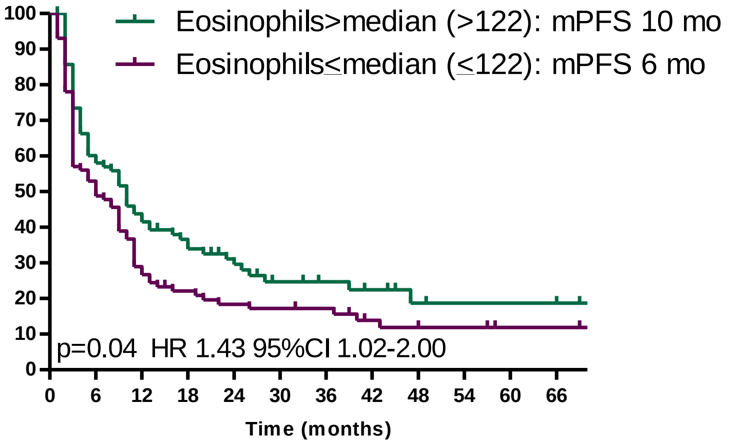

Months	0	6	12	18	24	30	36	42
EO>median	99	58	39	27	21	13	11	8
EO≤median	101	51	26	18	15	13	11	7

OS (%) all patients

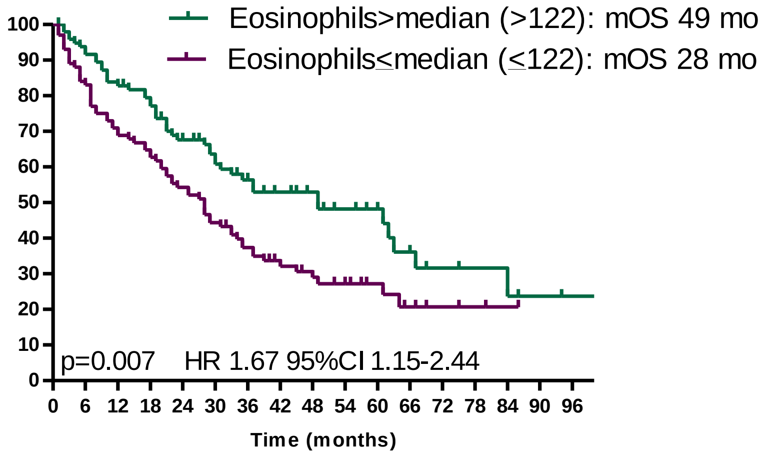

Months	0	6	12	18	24	30	36	42
EO>median	99	88	76	69	55	46	34	25
EO≤median	101	84	69	63	50	40	31	22

SUPPLEMENTAL FIGURE 6: ASSOCIATION OF NLR AND PFS/OS.

PFS (%) all patients

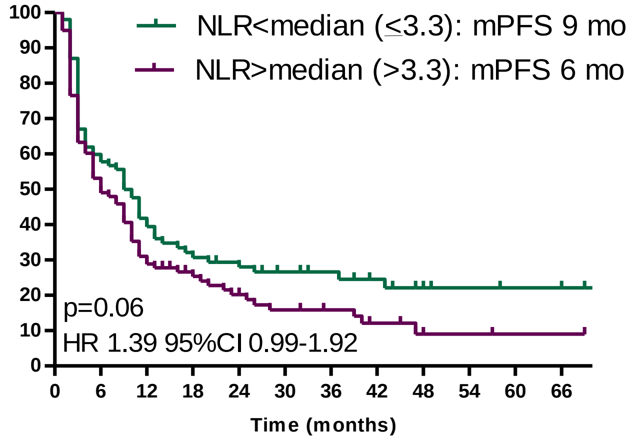

Months	0	6	12	18	24	30	36	42
NLR≤median	101	57	36	24	21	15	13	10
NLR>median	99	52	29	21	15	11	9	5

OS (%) all patients

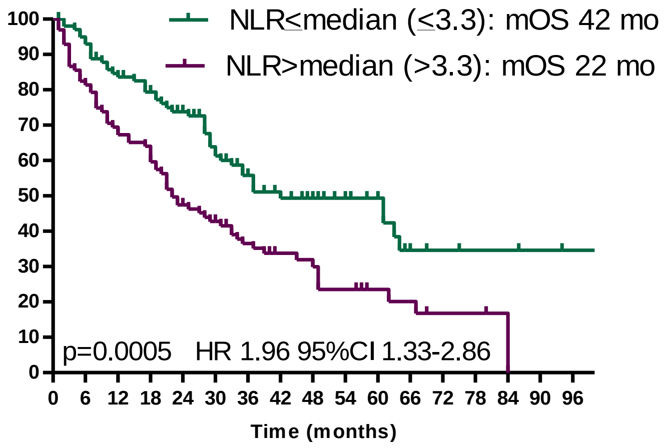

Months	0	6	12	18	24	30	36	42
NLR≤median	101	94	81	74	63	50	37	29
NLR>median	99	78	64	58	42	36	28	18

SUPPLEMENTAL FIGURE 7: PANEL A-C: KAPLAN-MEIER ESTIMATES OF THE IMPACT OF NER, NLR AND EOSINOPHILS ON DISEASE-FREE SURVIVAL POST-NEPHRECTOMY

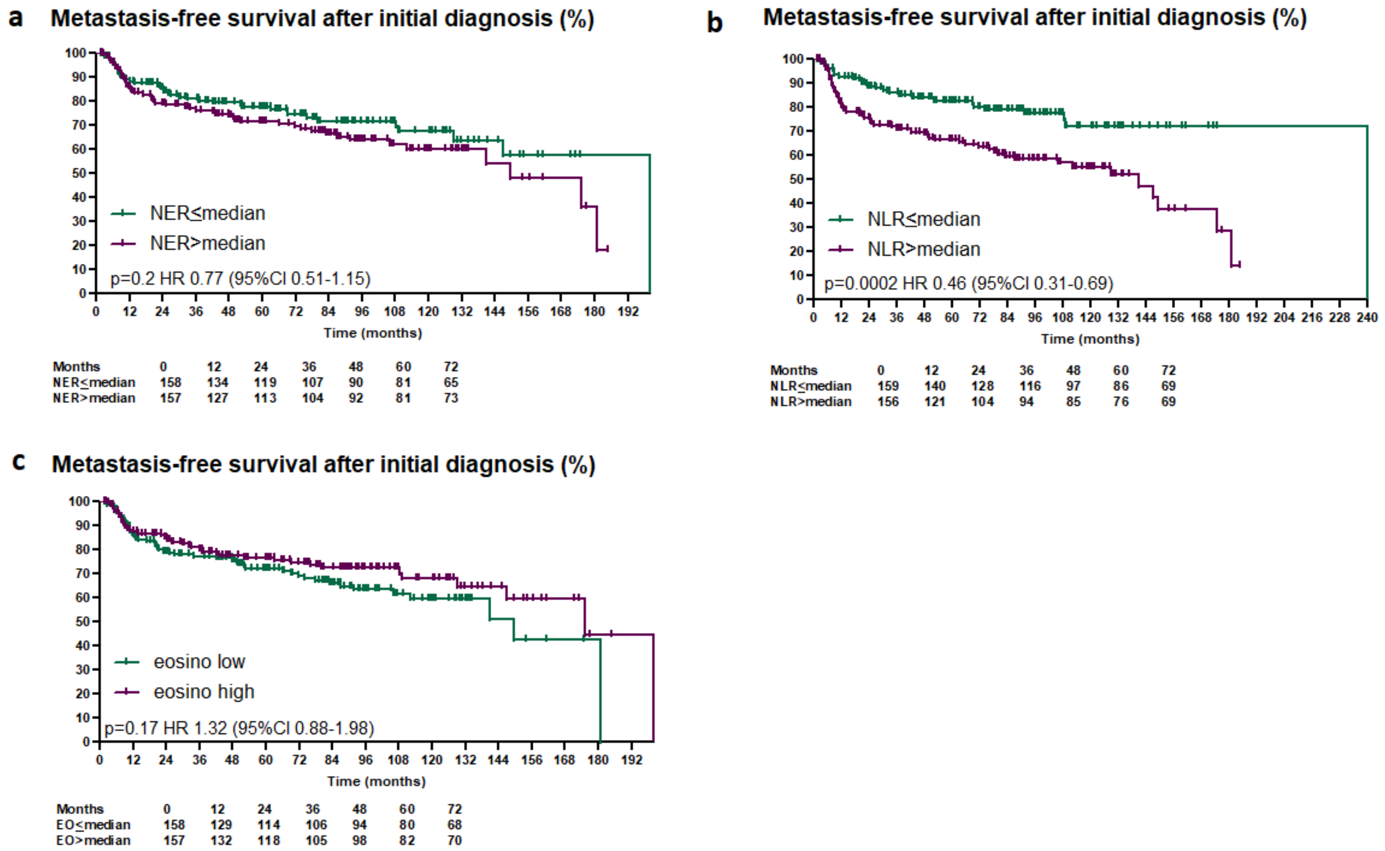

SUPPLEMENTAL FIGURE 8: ASSOCIATION OF MAXIMAL TUMOUR SHRINKAGE ON VEGFR-TKIS AND NER

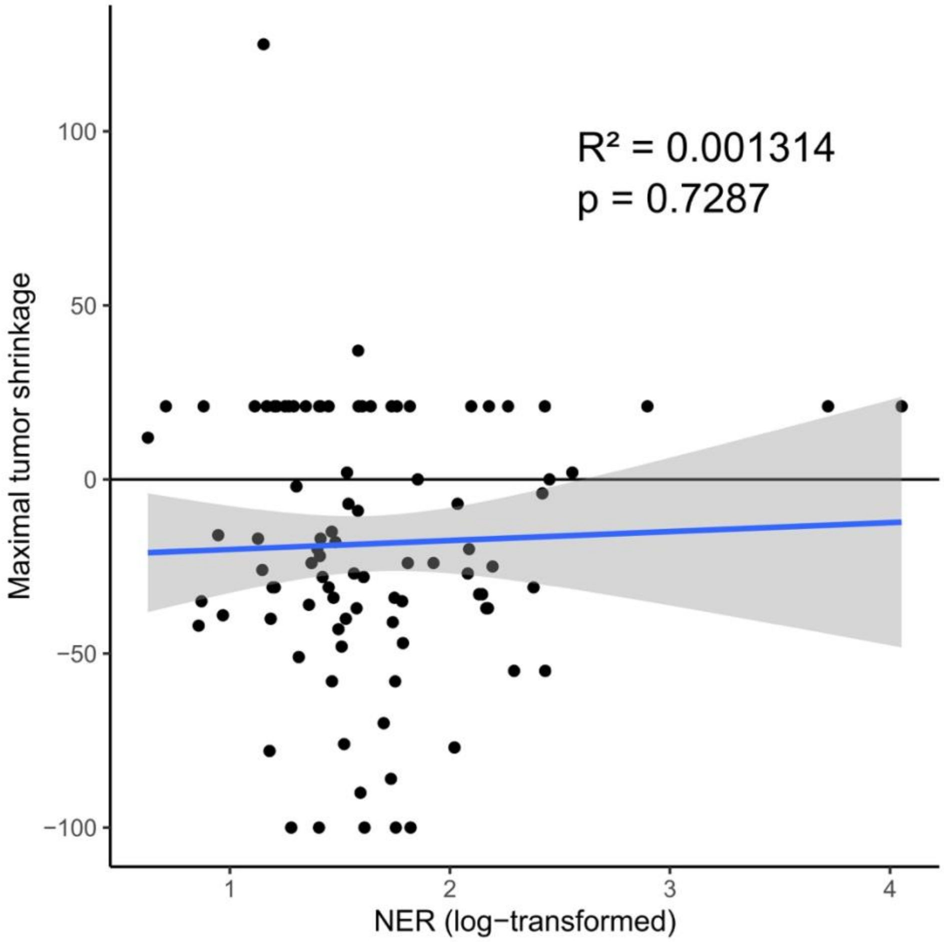

SUPPLEMENTAL FIGURE 9: PANEL A-B: KAPLAN-MEIER ESTIMATES OF THE IMPACT OF EOSINOPHILS ON PROGRESSION-FREE SURVIVAL AND OVERALL SURVIVAL IN ALL PATIENTS TREATED WITH VEGFR-TKIs

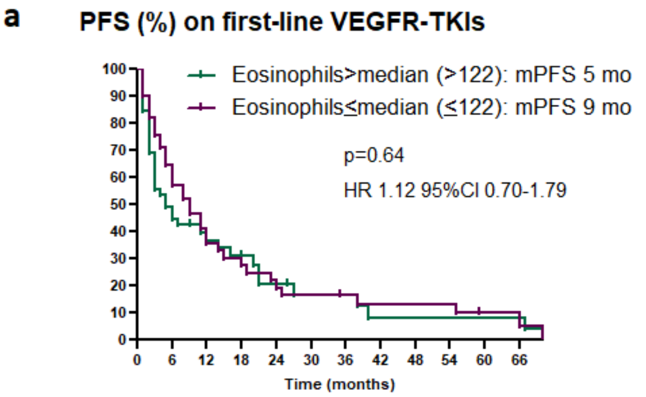

Months	0	6	12	18	24	30	36
EO>median	46	22	14	10	6	4	4
EO≤median	51	27	15	11	8	6	5

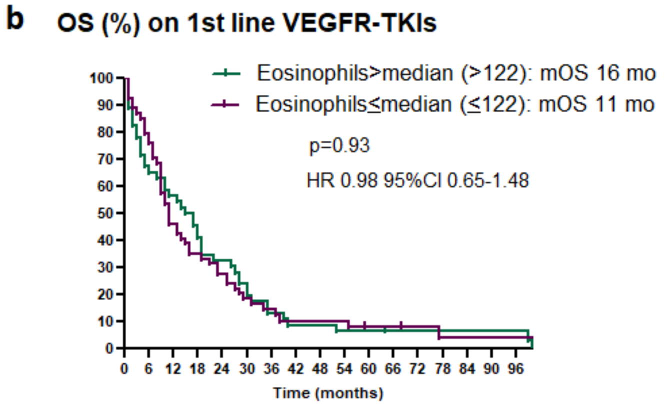

Months	0	6	12	18	24	30	36	42
EO>median	46	31	26	21	15	8	4	4
EO≤median	54	43	25	19	13	8	5	5

SUPPLEMENTAL FIGURE 10: PANEL A-B: KAPLAN MEIER ESTIMATES OF PFS AND OS BASED ON NER AT WEEK 6. PANEL C-D: KAPLAN MEIER ESTIMATES OF PFS AND OS BASED ON EOSINOPHIL COUNT AT WEEK 6.

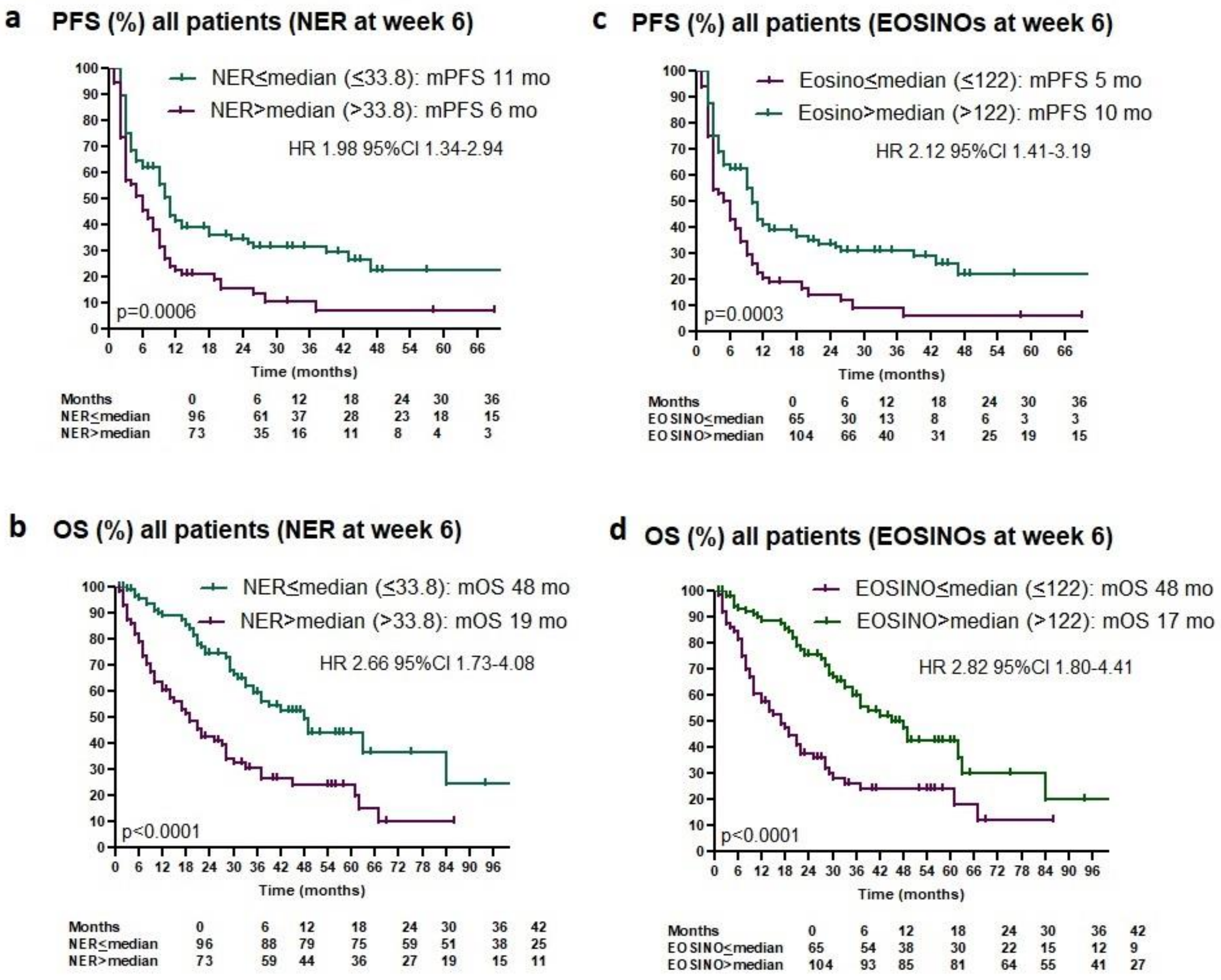

SUPPLEMENTAL FIGURE 11: Transcriptomic correlates of baseline eosinophil counts. PANEL A, Dotplot showing correlation coefficient and p values of spearman correlation between baseline eosinophil count and tumour immune cell estimates (CIBERSORTx). PANEL B: Dotplot showing correlation coefficient and p values of spearman correlation between baseline eosinophil count and PD-L1 (by *CD274* expression), tLHP and Javelin101 and IMmotion150 gene signatures. PANEL C: GSEA leveraging Hallmark gene sets, comparing patients with high vs. low eosinophil count (by median, also for panels D-F). PANEL D, GSEA leveraging Reactome gene sets. PANEL E, GSEA leveraging Gene Ontology BP gene sets. PANEL F, GSEA leveraging ImmuneSigDB gene sets. For panels D-F, only top 25 gene sets enriched in high eosinophil count and top 25 enriched in low eosinophil count are shown. NES: normalized enrichment score.

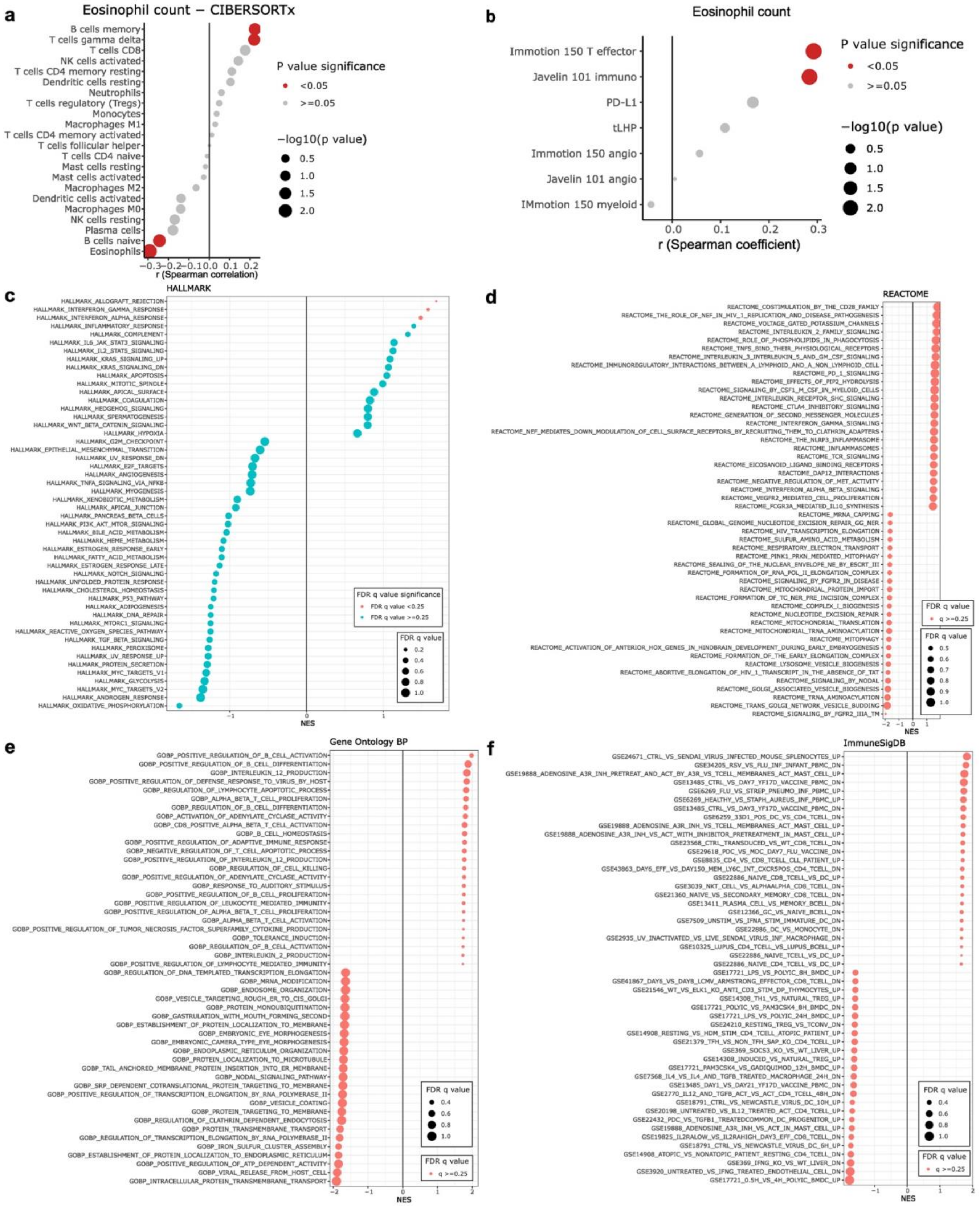

a
Eosinophil count – CIBERSORTx
B cells memory
T cells gamma delta
T cells CD8
NK cells activated
T cells CD4 memory resting
Dendritic cells resting
Neutrophils
T cells regulatory (Tregs)
Monocytes
Macrophages M1
T cells CD4 memory activated
T cells follicular helper
T cells CD4 naive
Mast cells resting
Mast cells activated
Macrophages M2
Dendritic cells activated
Macrophages M0
NK cells resting
Plasma cells
B cells naive
Eosinophils
−0.3 −0.2 −0.1 0.0 0.1 0.2
r (Spearman correlation)
P value significance
<0.05
>=0.05
−log10(p value)
0.5
1.0
1.5
2.0
b
Eosinophil count
Immotion 150 T effector
Javelin 101 immuno
PD-L1
tLHP
Immotion 150 angio
Javelin 101 angio
IMmotion 150 myeloid
0.0 0.1 0.2 0.3
r (Spearman coefficient)
P value significance
<0.05
>=0.05
−log10(p value)
0.5
1.0
1.5
2.0
c
HALLMARK
HALLMARK_ALLOGRAFT_REJECTION
HALLMARK_INTERFERON_GAMMA_RESPONSE
HALLMARK_INTERFERON_ALPHA_RESPONSE
HALLMARK_INFLAMMATORY_RESPONSE
HALLMARK_COMPLEMENT
HALLMARK_IL6_JAK_STAT3_SIGNALING
HALLMARK_IL2_STAT5_SIGNALING
HALLMARK_KRAS_SIGNALING_UP
HALLMARK_KRAS_SIGNALING_DN
HALLMARK_APOPTOSIS
HALLMARK_MITOTIC_SPINDLE
HALLMARK_APICAL_SURFACE
HALLMARK_COAGULATION
HALLMARK_HEDGEHOG_SIGNALING
HALLMARK_SPERMATOGENESIS
HALLMARK_WNT_BETA_CATENIN_SIGNALING
HALLMARK_HYPOXIA
HALLMARK_G2M_CHECKPOINT
HALLMARK_EPITHELIAL_MESENCHYMAL_TRANSITION
HALLMARK_UV_RESPONSE_DN
HALLMARK_E2F_TARGETS
HALLMARK_ANGIOGENESIS
HALLMARK_TNFA_SIGNALING_VIA_NFKB
HALLMARK_MYOGENESIS
HALLMARK_XENOBIOTIC_METABOLISM
HALLMARK_APICAL_JUNCTION
HALLMARK_PANCREAS_BETA_CELLS
HALLMARK_PI3K_AKT_MTOR_SIGNALING
HALLMARK_BILE_ACID_METABOLISM
HALLMARK_HEME_METABOLISM
HALLMARK_ESTROGEN_RESPONSE_EARLY
HALLMARK_FATTY_ACID_METABOLISM
HALLMARK_ESTROGEN_RESPONSE_LATE
HALLMARK_NOTCH_SIGNALING
HALLMARK_UNFOLDED_PROTEIN_RESPONSE
HALLMARK_CHOLESTEROL_HOMEOSTASIS
HALLMARK_P53_PATHWAY
HALLMARK_ADIPOGENESIS
HALLMARK_DNA_REPAIR
HALLMARK_MTORC1_SIGNALING
HALLMARK_REACTIVE_OXYGEN_SPECIES_PATHWAY
HALLMARK_TGF_BETA_SIGNALING
HALLMARK_PEROXISOME
HALLMARK_UV_RESPONSE_UP
HALLMARK_PROTEIN_SECRETION
HALLMARK_MYC_TARGETS_V1
HALLMARK_GLYCOLYSIS
HALLMARK_MYC_TARGETS_V2
HALLMARK_ANDROGEN_RESPONSE
HALLMARK_OXIDATIVE_PHOSPHORYLATION
-1 0 1
NES
FDR q value significance
FDR q value <0.25
FDR q value >=0.25
FDR q value
0.2
0.4
0.6
0.8
1.0
d
REACTOME
REACTOME_COSTIMULATION_BY_THE_CD28_FAMILY
REACTOME_THE_ROLE_OF_NEF_IN_HIV_1_REPLICATION_AND_DISEASE_PATHOGENESIS
REACTOME_VOLTAGE_GATED_POTASSIUM_CHANNELS
REACTOME_INTERLEUKIN_2_FAMILY_SIGNALING
REACTOME_ROLE_OF_PHOSPHOLIPIDS_IN_PHAGOCYTOSIS
REACTOME_TNFS_BIND_THEIR_PHYSIOLOGICAL_RECEPTORS
REACTOME_INTERLEUKIN_3_INTERLEUKIN_5_AND_GM_CSF_SIGNALING
REACTOME_IMMUNOREGULATORY_INTERACTIONS_BETWEEN_A_LYMPHOID_AND_A_NON_LYMPHOID_CELL
REACTOME_PD_1_SIGNALING
REACTOME_EFFECTS_OF_PIP2_HYDROLYSIS
REACTOME_SIGNALING_BY_CSF1_M_CSF_IN_MYELOID_CELLS
REACTOME_INTERLEUKIN_RECEPTOR_SHC_SIGNALING
REACTOME_CTLA4_INHIBITORY_SIGNALING
REACTOME_GENERATION_OF_SECOND_MESSENGER_MOLECULES
REACTOME_INTERFERON_GAMMA_SIGNALING
REACTOME_NEF_MEDIATES_DOWN_MODULATION_OF_CELL_SURFACE_RECEPTORS_BY_RECRUITING_THEM_TO_CLATHRIN_ADAPTERS
REACTOME_THE_NLRP3_INFLAMMASOME
REACTOME_INFLAMMASOMES
REACTOME_TCR_SIGNALING
REACTOME_EICOSANOID_LIGAND_BINDING_RECEPTORS
REACTOME_DAP12_INTERACTIONS
REACTOME_NEGATIVE_REGULATION_OF_MET_ACTIVITY
REACTOME_INTERFERON_ALPHA_BETA_SIGNALING
REACTOME_VEGFR2_MEDIATED_CELL_PROLIFERATION
REACTOME_FCGR3A_MEDIATED_IL10_SYNTHESIS
REACTOME_MRNA_CAPPING
REACTOME_GLOBAL_GENOME_NUCLEOTIDE_EXCISION_REPAIR_GG_NER
REACTOME_HIV_TRANSCRIPTION_ELONGATION
REACTOME_SULFUR_AMINO_ACID_METABOLISM
REACTOME_RESPIRATORY_ELECTRON_TRANSPORT
REACTOME_PINK1_PRKN_MEDIATED_MITOPHAGY
REACTOME_SEALING_OF_THE_NUCLEAR_ENVELOPE_NE_BY_ESCRT_III
REACTOME_FORMATION_OF_RNA_POL_II_ELONGATION_COMPLEX
REACTOME_SIGNALING_BY_FGFR2_IN_DISEASE
REACTOME_MITOCHONDRIAL_PROTEIN_IMPORT
REACTOME_FORMATION_OF_TC_NER_PRE_INCISION_COMPLEX
REACTOME_COMPLEX_I_BIOGENESIS
REACTOME_NUCLEOTIDE_EXCISION_REPAIR
REACTOME_MITOCHONDRIAL_TRANSLATION
REACTOME_MITOCHONDRIAL_TRNA_AMINOACYLATION
REACTOME_MITOPHAGY
REACTOME_ACTIVATION_OF_ANTERIOR_HOX_GENES_IN_HINDBRAIN_DEVELOPMENT_DURING_EARLY_EMBRYOGENESIS
REACTOME_FORMATION_OF_THE_EARLY_ELONGATION_COMPLEX
REACTOME_LYSOSOME_VESICLE_BIOGENESIS
REACTOME_ABORTIVE_ELONGATION_OF_HIV_1_TRANSCRIPT_IN_THE_ABSENCE_OF_TAT
REACTOME_SIGNALING_BY_NODAL
REACTOME_GOLGI_ASSOCIATED_VESICLE_BIOGENESIS
REACTOME_TRNA_AMINOACYLATION
REACTOME_TRANS_GOLGI_NETWORK_VESICLE_BUDDING
REACTOME_SIGNALING_BY_FGFR2_IIIA_TM
-2 -1 0 1
NES
FDR q value
q >=0.25
FDR q value
0.5
0.6
0.7
0.8
0.9
1.0
e
Gene Ontology BP
GOBP_POSITIVE_REGULATION_OF_B_CELL_ACTIVATION
GOBP_POSITIVE_REGULATION_OF_B_CELL_DIFFERENTIATION
GOBP_INTERLEUKIN_12_PRODUCTION
GOBP_POSITIVE_REGULATION_OF_DEFENSE_RESPONSE_TO_VIRUS_BY_HOST
GOBP_REGULATION_OF_LYMPHOCYTE_APOPTOTIC_PROCESS
GOBP_ALPHA_BETA_T_CELL_PROLIFERATION
GOBP_REGULATION_OF_B_CELL_DIFFERENTIATION
GOBP_ACTIVATION_OF_ADENYLATE_CYCLASE_ACTIVITY
GOBP_CD8_POSITIVE_ALPHA_BETA_T_CELL_ACTIVATION
GOBP_B_CELL_HOMEOSTASIS
GOBP_POSITIVE_REGULATION_OF_ADAPTIVE_IMMUNE_RESPONSE
GOBP_NEGATIVE_REGULATION_OF_T_CELL_APOPTOTIC_PROCESS
GOBP_POSITIVE_REGULATION_OF_INTERLEUKIN_12_PRODUCTION
GOBP_REGULATION_OF_CELL_KILLING
GOBP_POSITIVE_REGULATION_OF_ADENYLATE_CYCLASE_ACTIVITY
GOBP_RESPONSE_TO_AUDITORY_STIMULUS
GOBP_POSITIVE_REGULATION_OF_B_CELL_PROLIFERATION
GOBP_POSITIVE_REGULATION_OF_LEUKOCYTE_MEDIATED_IMMUNITY
GOBP_POSITIVE_REGULATION_OF_ALPHA_BETA_T_CELL_PROLIFERATION
GOBP_ALPHA_BETA_T_CELL_ACTIVATION
GOBP_POSITIVE_REGULATION_OF_TUMOR_NECROSIS_FACTOR_SUPERFAMILY_CYTOKINE_PRODUCTION
GOBP_TOLERANCE_INDUCTION
GOBP_REGULATION_OF_B_CELL_ACTIVATION
GOBP_INTERLEUKIN_2_PRODUCTION
GOBP_POSITIVE_REGULATION_OF_LYMPHOCYTE_MEDIATED_IMMUNITY
GOBP_REGULATION_OF_DNA_TEMPLATED_TRANSCRIPTION_ELONGATION
GOBP_MRNA_MODIFICATION
GOBP_ENDOSOME_ORGANIZATION
GOBP_VESICLE_TARGETING_ROUGH_ER_TO_CIS_GOLGI
GOBP_PROTEIN_MONOUBIQUITINATION
GOBP_GASTRULATION_WITH_MOUTH_FORMING_SECOND
GOBP_ESTABLISHMENT_OF_PROTEIN_LOCALIZATION_TO_MEMBRANE
GOBP_EMBRYONIC_EYE_MORPHOGENESIS
GOBP_EMBRYONIC_CAMERA_TYPE_EYE_MORPHOGENESIS
GOBP_ENDOPLASMIC_RETICULUM_ORGANIZATION
GOBP_PROTEIN_LOCALIZATION_TO_MICROTUBULE
GOBP_TAIL_ANCHORED_MEMBRANE_PROTEIN_INSERTION_INTO_ER_MEMBRANE
GOBP_NODAL_SIGNALING_PATHWAY
GOBP_SRP_DEPENDENT_COTRANSLATIONAL_PROTEIN_TARGETING_TO_MEMBRANE
GOBP_POSITIVE_REGULATION_OF_TRANSCRIPTION_ELONGATION_BY_RNA_POLYMERASE_II
GOBP_VESICLE_COATING
GOBP_PROTEIN_TARGETING_TO_MEMBRANE
GOBP_REGULATION_OF_CLATHRIN_DEPENDENT_ENDOCYTOSIS
GOBP_PROTEIN_TRANSMEMBRANE_TRANSPORT
GOBP_REGULATION_OF_TRANSCRIPTION_ELONGATION_BY_RNA_POLYMERASE_II
GOBP_IRON_SULFUR_CLUSTER_ASSEMBLY
GOBP_ESTABLISHMENT_OF_PROTEIN_LOCALIZATION_TO_ENDOPLASMIC_RETICULUM
GOBP_POSITIVE_REGULATION_OF_ATP_DEPENDENT_ACTIVITY
GOBP_VIRAL_RELEASE_FROM_HOST_CELL
GOBP_INTRACELLULAR_PROTEIN_TRANSMEMBRANE_TRANSPORT
-2 -1 0 1 2
NES
FDR q value
0.4
0.6
0.8
1.0
FDR q value
q >=0.25
f
ImmuneSigDB
-1 0 1 2
NES
FDR q value
0.4
0.6
0.8
1.0
FDR q value
q >=0.25

SUPPLEMENTAL FIGURE 12: Gene set enrichment analysis (GSEA) comparing NER^{HIGH} vs. NER^{LOW} groups (dichotomized by median). PANEL A, GSEA leveraging Hallmark gene sets. PANEL B, GSEA leveraging Reactome gene sets. PANEL C, GSEA leveraging Gene Ontology BP gene sets. PANEL D, GSEA leveraging ImmuneSigDB gene sets. For b-d, only top 25 gene sets enriched in NER^{HIGH} and top 25 enriched in NER^{LOW} are shown. NES: normalized enrichment score.

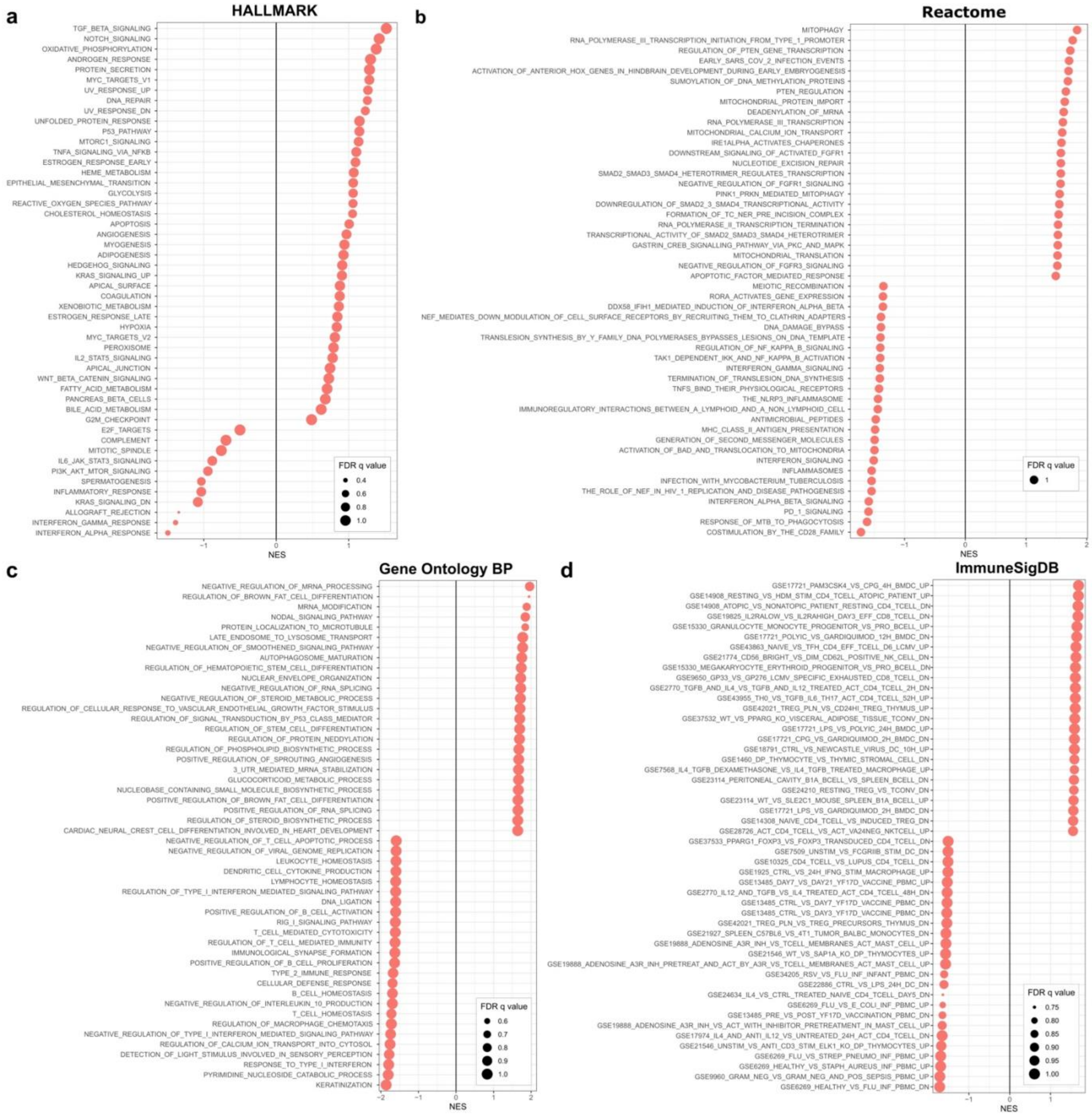

SUPPLEMENTAL FIGURE 13: Gene set enrichment analysis (GSEA) comparing NLR^HIGH vs. NLR^LOW groups (dichotomized by median). PANEL A, GSEA leveraging Hallmark gene sets. PANEL B, GSEA leveraging Reactome gene sets. PANEL C, GSEA leveraging Gene Ontology BP gene sets. PANEL D, GSEA leveraging ImmuneSigDB gene sets. For b-d, only top 25 gene sets enriched in NLR^HIGH and top 25 enriched in NLR^LOW are shown. NES: normalized enrichment score.

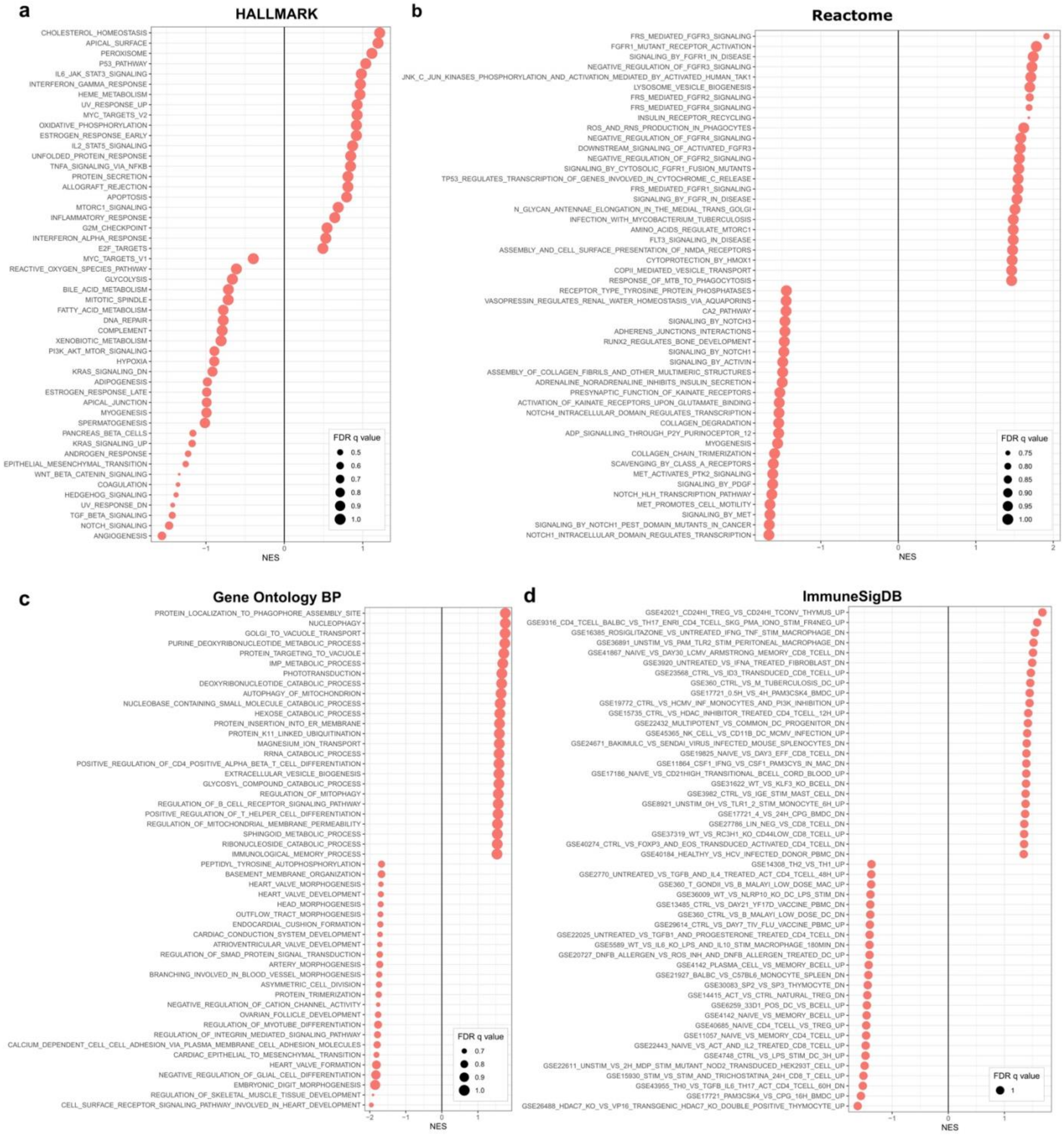

Supplement: Supplementary file 1 [file AO-63-40390-s1.pdf]
