# Supplementary material for: Baseline neutrophil-to-eosinophil-ratio and outcome in metastatic clear-cell renal cell carcinoma treated with nivolumab or ipilimumab/nivolumab
Source: Acta Oncol. 2024 Aug 11;63:40390. doi: 10.2340/1651-226X.2024.40390 (PMC11332468; doi:10.2340/1651-226X.2024.40390)
Supplement: Supplementary file 2 [file AO-63-40390-s2.pdf]

Supplementary material has been published as submitted. It has not been copyedited, or typeset by Acta Oncologica

SUPPLEMENTAL TABLE 1: HARREL C-INDEX

	C-index (95% CI)		
IMDC	IMDC + NER	IMDC + NLR	IMDC + EOSINO
**PFS**			
0.58 (0.54;0.63)	0.59 (0.54;0.65)	0.58 (0.53;0.64)	0.59 (0.54;0.65)
**OS**			
0.64 (0.60;0.69)	0.68 (0.63;0.73)	0.67 (0.61;0.73)	0.68 (0.63;0.73)

SUPPLEMENTAL TABLE 2: IMPACT OF NER IN OTHER CLINICAL SETTINGS

For NER, NLR and eosinophils, HR reflect the relative change in risk of progression or death for a doubling (x2 units) of the predictor value.

Test		HR (95% CI)	P-value
**Impact on time-to-metastasis (univariate analysis) (n=315)**			
NER	x2 units	1.12 (0.98;1.27)	0.10
NLR	x2 units	1.68 (1.28;2.20)	0.0002
Eosinophils	x2 units	0.92 ((0.79;1.06)	0.24
**Impact on PFS on VEGFR-TKIs (univariate analysis) (n=100)**			
NER	x2 units	1.22 (1.06;1.41)	0.007
Eosinophils	x2 units	0.86 (0.72;1.03)	0.09
**Impact on PFS on VEGFR-TKIs (bivariate analysis) (n=100)**			
NER	x2 units	1.13 (0.97;1.31)	0.11
IMDC	Global test		0.002
	Good vs Poor	0.23 (0.10;0.52)	0.0005
	Intermediate vs Poor	0.50 (0.29;0.87)	0.01
**Impact on OS on VEGFR-TKIs (univariate analysis) (n=100)**			
NER	x2 units	1.18 (1.05;1.32)	0.005
Eosinophils	x2 units	0.88 (0.76;1.01)	0.07
**Impact on OS on VEGFR-TKIs (bivariate analysis) (n=100)**			
NER	x2 units	1.11 (0.99;1.24)	0.08
IMDC	Global test		0.0003
	Good vs Poor	0.23 (0.11;0.48)	<.0001
	Intermediate vs Poor	0.48 (0.30;0.79)	0.004

SUPPLEMENTAL TABLE 3: IMPACT OF THE EVOLUTION OF NER AND EOSINOPHIL COUNT BETWEEN BASELINE AND WEEK 6

HR reflect the relative change in risk of progression or death for a doubling (x2 units) of the predictor value.

Variable	Test	HR (95% CI)	P-value
PFS (n=169)			
Change in NER	x2 units	1.04 (0.95;1.14)	0.36
Change in EO	x2 units	0.97 (0.88;1.07)	0.51
OS (n=169)			
Change in NER	x2 units	1.03 (0.93;1.14)	0.61
Change in EO	x2 units	0.97 (0.86;1.09)	0.62

SUPPLEMENTAL TABLE 4: SUMMARY OF LITERATURE DATA ON THE IMPACT OF NER ON OUTCOME

Article	Tumour type	Number of patients	Treatment	NER cut-off	OS	PFS	ORR
Zhuang et al. (2022)	mRCC	184	Nivolumab-Ipilumumab, Pembrolizumab, Avelumab	49.2	Univariate<br>HR 1.74<br>95%CI 1.11-2.74<br>p=0.02<br><br>Multivariate<br>HR 1.68<br>95%CI 1.01-2.82<br>p=0.05	Univariate<br>HR 1.23<br>95%CI 0.85-1.78<br>p=0.27<br><br>Multivariate<br>HR 1.37<br>95%CI 0.92-2.04<br>p=0.12	24.4% vs 21.9%.
Tucker et al. (2021)	m-ccRCC	110	Nivolumab-Ipilumumab	26.4<br>(=median)	Univariate<br>NR vs 27.3 months<br>HR 3.22<br>p<0.01<br><br>Multivariaat<br>HR 3.13<br>p<0.01	Univariate<br>8.6 vs 3.2 months<br>HR 2<br>p<0.01<br><br>Multivariaat<br>HR 1.61<br>p=0.04	40% vs 21.8%<br>OR 2.39<br>p=0.04
Gil et al. (2022)	mRCC	49	Nivolumab	48	Univariate<br>24 vs 6 months<br>p=0.002<br><br>Multivariate<br>HR 3.85<br>95%CI 1.33-11.17<br>p=0.013	Univariate<br>30 vs 3 months<br>p<0.001<br><br>Multivariate<br>HR 3.92<br>95%CI 1.66-9.23<br>p=0.002	87.5% vs 12.5%<br>p=0.003
Beulque et al.	m-ccRCC	201	Nivolumab or Nivolumab-Ipilumumab	33.8<br>(=median)	Univariate<br>HR 1.326<br>95%CI 1.20-1.47<br>p<0.0001<br><br>Multivariate<br>HR 1.21<br>95%CI 1.07-1.38<br>p=0.003	Univariate<br>HR 1.17<br>95%CI 1.06-1.29<br>p=0.002<br><br>Multivariate<br>HR 1.14<br>95%CI 1.01-1.28<br>p=0.04	37% vs 27%<br>p=0.17

m-ccRCC = metastatic clear cell renal cell cancer. HR = hazard ratio. NR = not reached
